# The Impact of Intramammary *Escherichia coli* Challenge on Liver and Mammary Transcriptome and Cross-Talk in Dairy Cows during Early Lactation Using RNAseq

**DOI:** 10.1371/journal.pone.0157480

**Published:** 2016-06-23

**Authors:** K. M. Moyes, P. Sørensen, M. Bionaz

**Affiliations:** 1 Department of Animal and Avian Sciences, University of Maryland, College Park, Maryland, United States of America; 2 Center for Quantitative Genetics and Genomics, Department of Molecular Biology and Genetics, Aarhus University, 8830 Tjele, Denmark; 3 Department of Animal and Rangeland Sciences, Oregon State University, Corvallis, Oregon, United States of America; University of Lleida, SPAIN

## Abstract

Our objective was to identify the biological response and the cross-talk between liver and mammary tissue after intramammary infection (IMI) with *Escherichia coli* (*E*. *coli*) using RNAseq technology. Sixteen cows were inoculated with live *E*. *coli* into one mammary quarter at ~4–6 weeks in lactation. For all cows, biopsies were performed at -144, 12 and 24 h relative to IMI in liver and at 24 h post-IMI in infected and non-infected (control) mammary quarters. For a subset of cows (n = 6), RNA was extracted from both liver and mammary tissue and sequenced using a 100 bp paired-end approach. Ingenuity Pathway Analysis and the Dynamic Impact Approach analysis of differentially expressed genes (overall effect False Discovery Rate≤0.05) indicated that IMI induced an overall activation of inflammation at 12 h post-IMI and a strong inhibition of metabolism, especially related to lipid, glucose, and xenobiotics at 24 h post-IMI in liver. The data indicated in mammary tissue an overall induction of inflammatory response with little effect on metabolism at 24 h post-IMI. We identified a large number of up-stream regulators potentially involved in the response to IMI in both tissues but a relatively small core network of transcription factors controlling the response to IMI for liver whereas a large network in mammary tissue. Transcriptomic results in liver and mammary tissue were supported by changes in inflammatory and metabolic mediators in blood and milk. The analysis of potential cross-talk between the two tissues during IMI uncovered a large communication from the mammary tissue to the liver to coordinate the inflammatory response but a relatively small communication from the liver to the mammary tissue. Our results indicate a strong induction of the inflammatory response in mammary tissue and impairment of liver metabolism 24h post-IMI partly driven by the signaling from infected mammary tissue.

## Introduction

During early lactation (i.e. the first 60 days of lactation), the massive repartition of nutrients to the mammary gland for milk synthesis has been identified as a major contributor to the high risk of developing diseases [1]. This repartition of energy toward the mammary gland is not compensated via feed intake, that is also reaching a nadir during early lactation [1]. The requirement of energy and nutrients increases ~5-fold from pregnancy to lactation in high producing dairy cows mainly due to the large amount of milk synthesized by the mammary gland [2]. In order to meet the nutrient demands in early lactation, most cows mobilize body tissue, e.g. skeletal muscle and adipose tissue, and thereby experience a period of negative energy balance, as reflected by the degree of increase in circulating non-esterified fatty acids (**NEFA**), ketone bodies (β-hydroxybutyrate; **BHBA**) and decrease in blood glucose [3]. As a result, production diseases, such as ketosis and hepatic lipidosis, occur most often at this time [4] and are associated with negative impacts on animal health and reduced economic outcome to the farmer.

The liver plays a central role in the metabolic and inflammatory physiology of the dairy cow. Dairy cows, being ruminants, have a negligible amount of glucose absorbed from the intestine [4]; therefore, the large amount of glucose needed to synthesize milk lactose is coming largely from the hepatic gluconeogenesis. Especially in early lactation, the liver is naturally compromised via increased gluconeogenesis and the catabolism of infiltrating NEFA. Besides its vital role in metabolism, the liver participates to the immune response by synthesizing and secreting into the bloodstream inflammatory mediators (i.e. acute phase proteins) [5]. Acute phase proteins are non-specific innate immune components involved in restoring homeostasis and providing host protection from invading microorganisms and inflammation [6]. Few studies have focused on the metabolic changes that occur in the liver after an IMI. Recent work has demonstrated a large transcriptomic response of the bovine liver after intramammary infection (**IMI**) challenge with **lipopolysaccharides (LPS**), an endotoxin released from the cell wall of *Escherichia coli* (***E*. *coli***) [7]. Results indicated an increase in the hepatic expression of transcripts coding for proteins involved in acute phase reaction (and other inflammatory related proteins) and a consequent/concomitant reduction in the expression of transcripts coding for key metabolic enzymes, including the ones involved in gluconeogenesis [7]. Therefore, during inflammatory states such as mastitis, the immunometabolic demands may compromise liver function and increase risk of disease.

The cross-talk between different tissues is emerging as an important regulatory factor during health and disease. This has been observed in monogastrics [8]. Mastitis, an inflammation of the mammary gland, is the most costly disease in the dairy industry and occurs more frequently during early lactation [9]. Mastitis is characterized by an increase in milk somatic cell count (**SCC**) and may be accompanied by the presence of an intramammary pathogen [10], such as *E*.*coli*, one of the most common and costly mastitis-causing pathogens [11]. The mammary tissue is highly dependent on a highly functional liver and, therefore, it appears reasonable that a direct cross-talk between the mammary gland and the liver exists to coordinate the nutrient demands for lactation and to elicit the immune response to mastitis.

Our objective was to characterize the individual response and the cross-talk between liver and mammary tissue in response to IMI with *E*. *coli* using RNAseq technology.

## Materials and Methods

Experimental procedures involving animals were approved by the Danish Animal Experiments Inspectorate and complied with the Danish Ministry of Justice Laws concerning animal experimentation and care of experimental animals.

### Animals, Experimental Design and Sample Collection

The animal trial was conducted at the Aarhus University’s dairy barn, Ammitsbøl Skovgaard (Denmark). Sixteen healthy primiparous Holstein cows at ~4–6 weeks in lactation were used for this study. The experimental design has been illustrated previously [12]. Cows were not treated for any clinical signs of disease before the study period. Details on animal housing, total mixed ration fed, treatment, preparation and inoculation of *E*. *coli* and clinical examinations have been previously described [13,14]. Briefly, cows were considered healthy and free of mastitis-causing pathogens based on body temperature, white blood cell count, glutaraldehyde test, California Mastitis Test (Kruuse, Marslev, Denmark) and bacteriological examinations of aseptic quarter foremilk samples prior to the start of the study period. Using the portable DeLaval Cell Counter (DeLaval, Tumba, Sweden), the front quarter with the lowest SCC (<27,000 cells/mL) was used for *E*. *coli* infusion. All eligible cows were inoculated with ~20–40 cfu of live *E*. *coli* (Danish field isolate k2bh2) into one front mammary quarter immediately following the afternoon milking (h = 0). Healthy, control quarters were chosen based upon bacteriological examinations where quarter SCC were <181,000 cells/mL at 24 h post-IMI. Daily feed intake and milk yield at each milking were recorded. Rectal temperature was recorded periodically and composite milk samples were collected at -180, -132, -84, -36, -12 h, 0, 12, 24, 36, 48, 60, 72 and 84 h relative to IMI challenge from infected quarters for analysis of lactate dehydrogenase (**LDH**), alkaline phosphatase (**AP**) and N-acetyl-β-D-glucosaminidase (**NAGase**) following procedures reported previously [15]. Aseptic quarter foremilk samples were collected at -12, 0, 3, 6, 12, 18, 24, 48, 60 and 84 h relative to IMI challenge from infected quarters. One day prior to IMI challenge, sterile Micro-Renathane polyvinyl catheters were inserted into the jugular vein and flushed with a sterile 0.9% NaCl solution containing 50 IU Na-heparin as previously described [12]. Blood was collected at -12, 0, 3, 6, 12, 18, 24, 36, 60 and 84 h relative to IMI challenge and plasma was analyzed for concentrations of glucose, NEFA, BHBA and cholesterol using an autoanalyzer, ADVIA 1650^®^ Chemistry System (Siemens Medical Solution, Tarrytown, NY, USA) as previously described [16]. Liver biopsies were collected from all cows at -144, 12 and 24 h relative to IMI challenge were analyzed for gene expression as previously described [17]; and, using a minimally invasive biopsy technique, mammary tissue was collected at 24 relative to IMI challenge from both infected and control quarters for gene expression analysis as previously described [13]. Combined biopsy had no effect on the production and inflammatory mediators presented for this study [12]. After the mammary biopsies had been collected, cows were administered a prophylactic antibiotic treatment against infection with Gram-positive bacteria by intramuscular injection of 30 mL of Penovet^®^ vet (300,000 IE benzylpenicillinprocain/ml; Boehringer Ingelheim Danmark A/S,Copenhagen, Denmark). No other antibiotic therapy was administered after IMI challenge.

### RNAseq Analysis

For a subset of liver and mammary tissue samples (n = 6 cows for both liver and mammary tissue), RNA extraction was performed as described previously [18]. RNA was extracted from the liver of 6 cows at 3 different time points (i.e. -144, 12 and 24 h relative to IMI challenge; 3 x 6 = 18 liver RNA samples in total) and for the mammary tissue from the infected and control quarters of the same 6 cows (i.e. 2 x 6 = 12 mammary tissue RNA samples in total). Each sample was sequenced using a 100 bp paired approach with the Illumina Hiseq2000 sequencing technology by AROS Applied Biotechnology (Aarhus, Denmark). The standard Illumina TruSeq mRNA protocol was used with minor changes (fragmentation time was 1 minute and the number of PCR cycles was 13). Sequence reads obtained for each sample were aligned separately to the Bovine genome assembly (UMD3.1). Sequence alignment was conducted using an efficient and sensitive mapping paradigm based on seed-and-vote algorithm [19] implemented in the Rsubread package in R. The abundance of mRNAs for all genomic features (bovine Ensembl genes) was calculated for each sample. Procedures previously described [20] were used to obtain the annotated bovine Ensembl genes from the annotations available at the UCSC Genome Browser. The total number of bovine Ensembl genes was 24,616. The function ‘Feature Counts’ in the R package was used to count the number of reads that mapped to each feature using the default settings. The total number of mapped reads for each sample varied from 10,434,369 to 22,098,317.

### Statistical Analysis

#### RNAseq

Differential expressed genes were identified using edgeR (version 3.4.2; [21]). The count data were normalized using the weighted trimmed mean of M-values [21]. Count data were non-normally distributed thus a generalized linear model (**GLM**) was fitted for each gene. A negative binomial GLM was used allowing for a quadratic mean-variance relationship commonly observed in this type of data. The GLM allows for a design to be modeled and differential expression is determined using a likelihood ratio test. In the GLM model specified the number reads in RNA sample i that map to the gene g is denoted y_gi_ and the total number of mapped reads is denoted N_i_. It is assumed that y_gi_ ∼ *NB*(μ_gi_, *ϕ*_*g*_), where μ_gi_ is the mean and *ϕ*_*g*_ is the dispersion parameters of the negative bionomial distribution. The expected number of reads for a particular gene μ_gi_ is the product of the relative abundance of that gene λ_gi_ and the total number of reads N_i_ in the i^th^ sample. It is assumes that λ_gi_ can be represented by a log-linear model:

$$\text{log}\left(\lambda_{gi}\right)=x_{i}^{T}\beta_{g}$$

where $x_{i}^{T}$ is the covariate vector indicating the treatment conditions applied to sample i and *β*_*g*_ is the vector of regression coefficients by which the covariate effects are mediated for gene g.

Liver and mammary tissue samples were analyzed separately. In the statistical analyses we only considered the main effect of time in case of liver samples (i.e. *β*_*g*_ has three treatment levels, -144, 12 and 24 h relative to IMI challenge) and infection status in case of mammary tissue samples (i.e. *β*_*g*_ has two treatment levels, Infected and Control). The aim of the differential expression analysis is to test the null hypothesis (**H**_**0**_) (stating that the relative abundance of reads for the gene g is similar in treatment condition *T*_1_ (e.g. time = -144hours) and *T*_2_ (e.g. time = 12hours)): $H_{0}:\lambda {\;}_{g}^{T_{1}}=\lambda {\;}_{g}^{T_{2}}$ against the alternative hypothesis **(H**_**1**_**)** (stating that the relative abundance of reads for the gene g is different in treatment condition *T*_1_ and *T*_2_): $H_{1}:\lambda {\;}_{g}^{T_{1}}\neq \lambda {\;}_{g}^{T_{2}}$. This corresponds to test the null hypothesis ${\text{H}}_{0}:\beta_{g}^{T1}=\beta_{g}^{T2}$ in the log-linear model which can be specified by a contrast vector *c*^*T*^ for H_0_: *c*^*T*^ *β*_*g*_ = 0. These hypotheses are evaluated by comparing the observed likelihood ratio test statistics to a *χ*^2^ distribution with 1 degree of freedom. For the liver we determined if there is differential expression between any of the time points (e.g. a joint test for the three comparisons: -144 vs 12, -144 vs 24 and 12 vs 24) or at specific time points (e.g. three individual tests one for each of comparison -144 vs 12, -144 vs 24 and 12 vs 24). For the mammary tissue determined if there is differential expression between the infected and uninfected quarter (e.g. a single test). To ensure stable inference for each gene an empirical Bayes method was applied to squeeze the gene wise dispersions towards a common dispersion for all genes [22]. As there are 24,616 genes annotated in the bovine genome, the statistical tests in each analysis were corrected for multiple testing using the Benjamini and Hochberg False Discovery Rate (**FDR**) method [23] as implemented in R (version 2.12.0).

The RNAseq data files were deposited in NCBI’s Gene Expression Omnibus (GEO; http://www.ncbi.nlm.nih.gov/geo/) and are accessible through GEO series accession number [GSE75379; http://www.ncbi.nlm.nih.gov/geo/query/acc.cgi?token=czgzgcwwfxwjtub&acc=GSE75379).

#### Blood and milk

The PROC MIXED procedure of SAS (SAS/STAT version 9.2; SAS Institute Inc., Cary, NC) was used for statistical analysis for all biopsied cows (n = 16). The class variables included cow, block (i.e. date of IMI challenge), and time (i.e. relative to IMI) and the model included the fixed effect of time and the random effect of cow nested within block. The degrees of freedom were estimated with the Kenward-Roger specification in the model statement. The data are presented as least squares mean (**LSM**) and the standard error of the mean (**SEM**). Statistical differences were declared as significant if *P* ≤ 0.05.

### Bioinformatics Analyses Using Ingenuity Pathway Analysis (IPA)

Network, function, and pathway analyses were generated using IPA (Ingenuity Systems, www.ingenuity.com) that assists with RNAseq data interpretation *via* grouping differentially expressed genes (**DEG**) into known functions, pathways, and networks based primarily on human and rodent studies. The whole dataset containing Ensembl Gene ID, FDR, expression ratio, and *P*-value for all comparisons was uploaded into IPA and the whole bovine annotated genome (24,616 unique Ensembl IDs) was used as background. Only 15,148 unique genes were mapped into IPA. Due to the nature of the sample analyzed the analysis carried out with IPA was restricted to IPA database related to liver as organ system and all Hepatoma cell lines for the data related to liver and IPA dataset related to mammary tissue as organ system and Brest Cancer cell lines and Other cell lines for the data related to mammary tissue. The genes used in the IPA were selected based on the following criteria: In the liver a FDR adjusted p-value below 0.05 for the joint test determining differentially expression at any of the time points, a fold change larger than 1 (or smaller than -1), and a p-value below 0.05 for each of the comparison -144 vs 12, -144 vs 24 and 12 vs 24. In the mammary tissue a FDR adjusted p-value below 0.05 for test comparing the infected and uninfected quarter. Both up- and down-regulated genes were analyzed simultaneously.

The IPA was used to analyze the up-stream regulators of DEG using the “Up-stream analysis” feature. The analysis uses an IPA Knowledge base to predict the expected causal effects between up-stream regulators and DEG targets. The analysis provides the more plausible prediction of the status of the up-stream regulator (i.e., activated or inhibited) by computing an overlap *P*-value and an activation z-score. The results were downloaded and graphically depicted using SigmaPlot v11 (Systat Software Inc., Germany).

Network analysis of DEG coding for transcriptional factors (**TF**; i.e., transcriptional network analysis) was performed using IPA. The network analysis allows to uncover (and to visualize) the interaction between TF and also provides an overall prediction of the TF activation/inhibition using expression of putative down-stream genes. Furthermore, the most significantly enriched functions were uncovered among DEG TF in the network using IPA.

The cross-talk between liver and mammary tissue at 24 h after *E*.*coli* IMI was performed using the network capability of IPA as previously described [24]. For the present analysis DEG were identified using as criteria: FDR < 0.05 for the overall effect (overall time effect for liver encompassing 12 and 24 h post-IMI and IMI vs. control for mammary tissue), an expression ratio ≥ 2, and *P*-value < 0.001; a higher stringency compared to the functional analysis was used in order to identify the genes coding for proteins with a higher likelihood of cross-talk between the tissues. The genes considered to code for secreted proteins were the one in the cytokines and growth factors categories while genes coding for proteins considered to be receptors that might be able to “sense” the secreted proteins were the one in G-protein coupled receptor, ligand-dependent nuclear receptor, transcription regulator, and transmembrane receptor. Networks between DEG with high expression in liver of cows at 24 h and coding for secreted proteins and DEG more expressed in *E*. *coli* treated vs. saline in mammary tissue and coding for receptors and *vice versa* were built using IPA Knowledge base.

### Bioinformatic Analyses Using Dynamic Impact Approach

The description of the Dynamic Impact Approach (**DIA**) tool is reported elsewhere [25]. For the DIA analysis, the Ensembl IDs were transformed into Entrez Gene ID using BioDBnet [26]. Only 16,867 unique Entrez Gene IDs were obtained and used for the analysis. The whole annotated dataset was uploaded into the system using the whole bovine annotation as background. As for IPA a FDR < 0.05 for the overall time effect and a *P*-value < 0.05 between comparisons were used as cut-off. The DIA analysis was performed for the Kyoto Encyclopedia of Genes and Genomes (**KEGG**) pathways [27].

## Results and Discussion

### Responses in Blood and Milk after IMI with *E*. *coli*

For all biopsied cows (n = 16), clinical symptoms and rises in milk SCC, shedding of *E*. *coli*, rectal temperature, heart rate and respiration were observed and results are reported elsewhere [12,15]. Change in concentration of AP, NAGase and LDH in milk after IMI with *E*. *coli* for all 16 cows is shown in S1 Fig. Alkaline phosphatase increased by 48 h after IMI when compared to pre-IMI levels (h = 0). Milk NAGase was elevated at 36, 48 and 72 h post-IMI. Milk LDH was significantly elevated at 36 h post-IMI whereas no other time points differed from h = 0. Our data clearly indicate an inflammatory response to IMI challenge with *E*. *coli*.

Feed intake and milk yield response for cows (n = 16) after IMI challenge with *E*. *coli* are shown in S2 Fig. Feed intake decreased by 48 h post-IMI and returned to pre-IMI levels by 72 h post-IMI whereas milk yield decreased by 24 h, remained lower through 48 h, and returned to pre-IMI levels by 60 h post-IMI. The pro-inflammatory response and the changing hormonal environment contribute to reduced feed intake and milk production observed during an IMI [28] and most likely explains the majority of variation in feed intake and milk yield observed for this study. Changes in plasma concentrations of NEFA, BHBA, glucose and cholesterol (n = 16) after IMI with *E*. *coli* are shown in S3 Fig. Overall, plasma NEFA increased during IMI but no differences were observed at any given time point. Elevated NEFA in blood indicates increased adipose tissue lipolysis during the inflammatory response [29]. Glucose concentrations increased by 36 and 60 h after IMI whereas plasma BHBA decreased by 12 h post-IMI and remained lower throughout the study period. Concentration of plasma cholesterol decreased and was significantly lower at 84 h post-IMI when compared to 0 h. Increases in plasma glucose are not associated with increased hepatic gluconeogenesis, impaired during periods of inflammation [30], but is likely consequence of insulin resistance typically induced by inflammation leading to hyperglycemia [31]. Drops in plasma BHBA can be primarily associated with increased transfer into milk [15]. Blood BHBA has been associated with reduced neutrophil recruitment [32] and may partly explain reduced levels of blood BHBA during inflammation. Changes in metabolites in blood and animal production during IMI are similar to those reported by others [33,34] and may increase risk for the development of subsequent disease during mastitis for dairy cows in early lactation.

### Transcriptome Altered in Liver and Mammary During IMI

**Fig 1:**
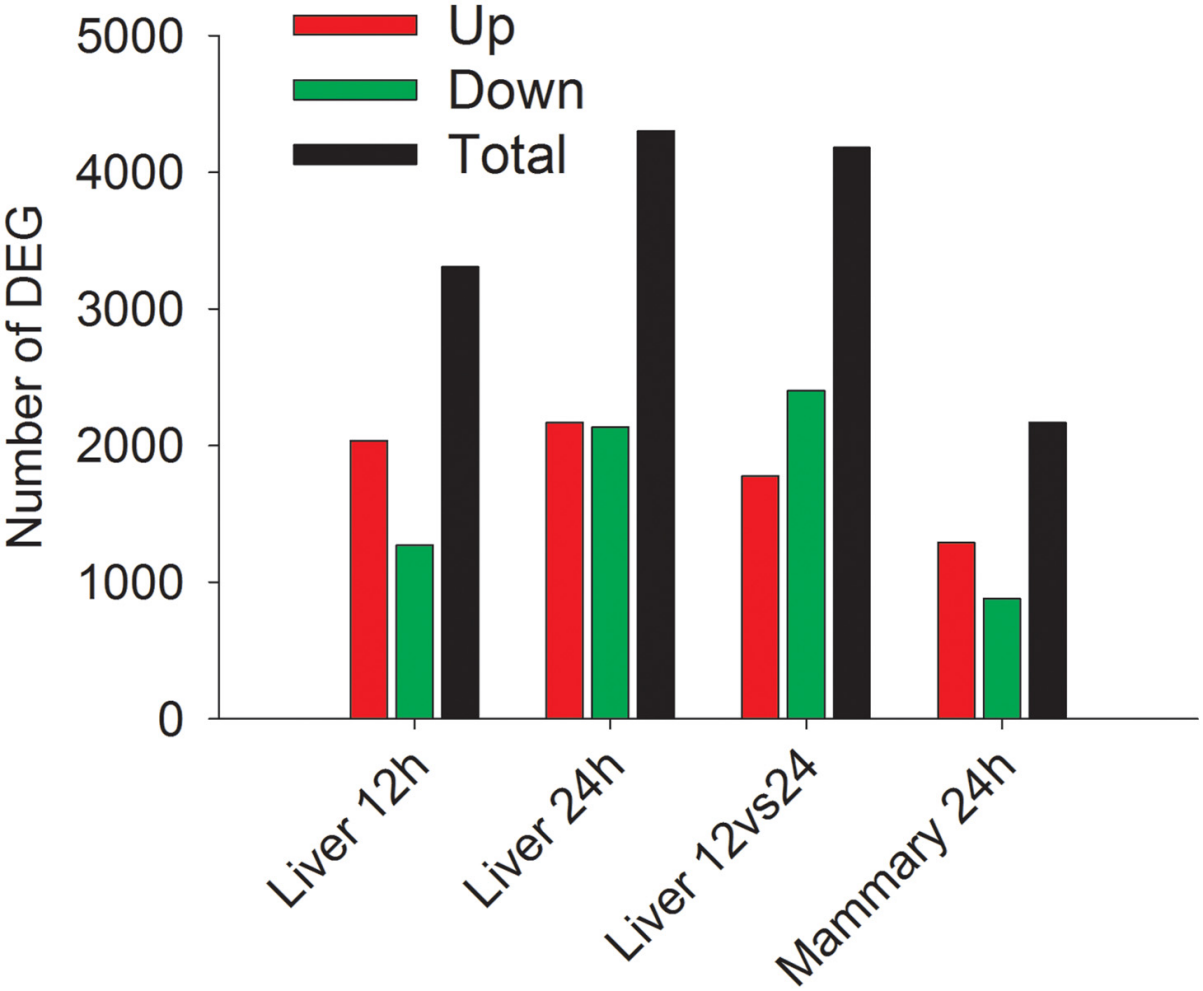
Number of Differentially Expressed Genes (DEG) in each comparison. The number of DEG (FDR <0.05, *P*-value between comparisons <0.05) as total (black), up-regulated (Up; red), and down-regulated (Down; green) in bovine liver at 12 and 24 h vs. -144 h relative to IMIchallenge with *E*. *coli* and at 24 vs. 12 h post-IMI. Reported is also the number of DEG between infected and non-infected bovine mammary quarters at 24 h post-IMI.

The complete dataset with statistical results is available in S5 Fig. Genomic analysis via RNAseq uncovered >3,643 and >4,724 DEG (Fig 1) in liver tissue at 12 and 24 h post-challenge compared to -144 h, respectively. By 12 h (i.e. early inflammatory response), 1,399 DEG were down-regulated and 2,244 were up-regulated whereas the same number of up- and down-regulated (2,388 up- and 2,336 down-regulated) were detected at 24 h post-challenge (i.e. peak inflammatory response) compared to 144 h pre-challenge. For mammary tissue, >2,379 DEG were observed 24 h post-challenge in the IMI compared to the control quarter with, similar to the liver at 12 h, more up-regulated (1,407) compared to down-regulated (972) DEG. Overall, the liver had a larger transcriptomic response to IMI when compared to mammary tissue. However, the mammary gland having a single comparison the use of a FDR cut-off for the overall effect of the treatment has accounted for the false positive while for the liver the false positives were accounted for the overall effect (i.e., time) but we did not corrected for false positive for the specific comparisons (i.e., 12 vs. 144 h, 24 vs. 144 h, and 24 vs. 12 h). The use of FDR ≤ 0.05 for the mammary tissue resulted in a *P*-value ≤ 0.005 (S5 Fig). The use of the same *P*-value cut-off for the liver resulted in lower DEG for the comparisons, with the comparison 12 vs. 144 h having 2,555 DEG and the comparison 24 vs. 144 h having 3,323 DEG (approx. 28% less DEG compared with the use of *P*-value ≤ 0.05). Thus, the overall transcriptomic effect of IMI was still larger for the liver compared to mammary tissue. Because of the difference in the overall effect considered for the mammary tissue (IMI vs. control) and the liver (time effect), we decided to use a consistent FDR for the overall effect and a *P*-value ≤ 0.05 for the various comparisons for the liver, while for the mammary the *P*-value was not used (but only the FDR for the overall effect). The use of a simple *P*-value (i.e. not corrected for multiple comparisons) ≤ 0.05 in liver is a liberal approach and can be a limitation when evaluating the functional analysis in enrichment approach analyses, such as the approach used by IPA. However, the *Z*-score for the up-stream regulator analysis in IPA can benefits from a larger number of DEG and the DIA is minimally affected by the *P*-value cut-off and, as for the *Z*-score, the algorithm benefits from a larger number of DEG. Thus, we opted for a more liberal *P*-value between comparisons in liver.

### Functional Analysis of the Liver Transcriptome: Early Induction of Inflammation followed by Large Inhibition of Metabolism

#### Hepatic transcriptomic response at 12 h post-IMI

**Fig 2:**
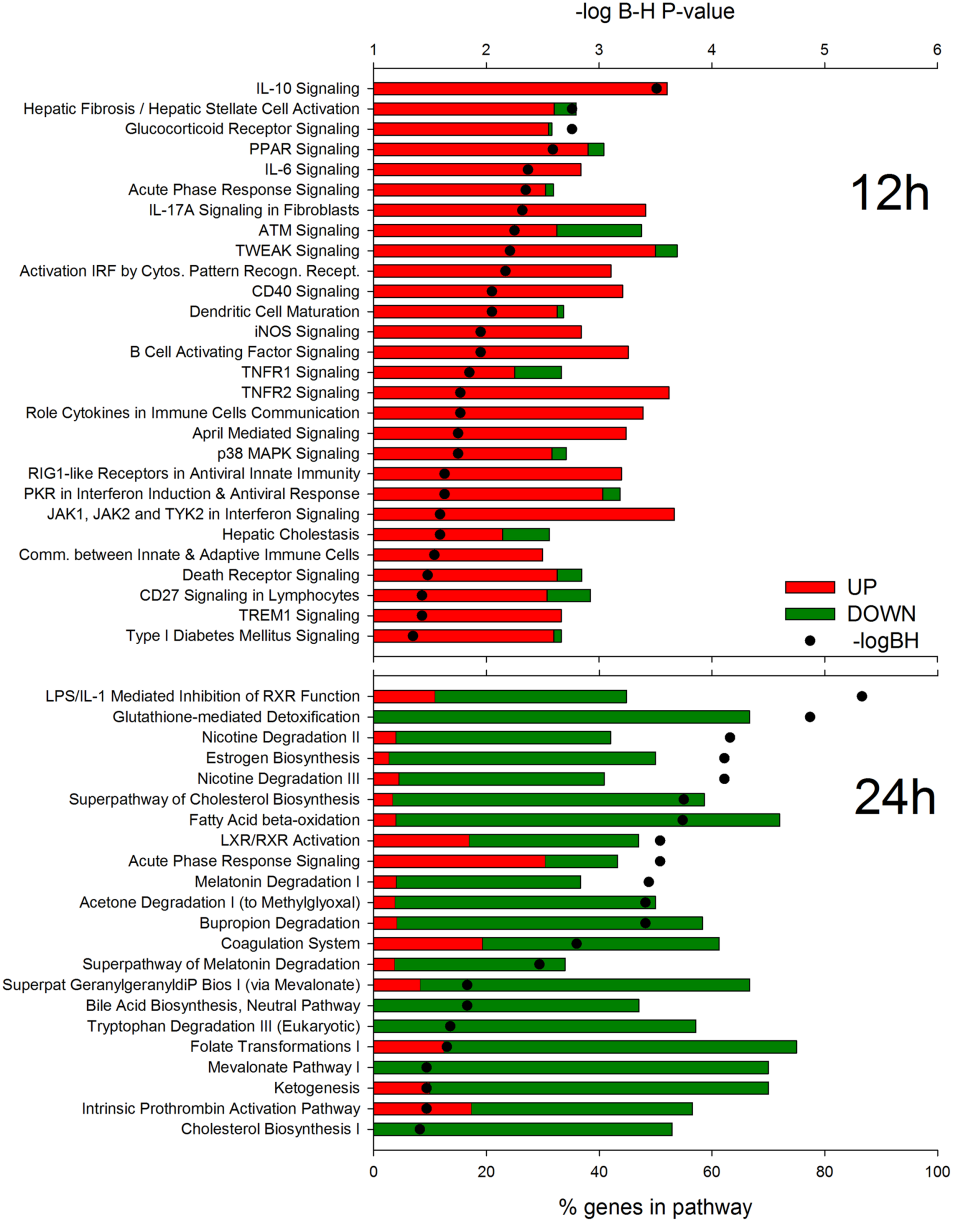
Most enriched pathways in liver at 12 and 24 h after intramammary infection (IMI) challenge with *E*. *coli*. Most enriched pathways among differentially expressed genes (DEG) in bovine liver at 12 and 24 vs. -144 h relative to IMI as uncovered by Ingenuity Pathway Analysis. Reported are the significance of enrichment (as–log_10_ of the Hochberg-Benjamini false discovery rate [-logBH; >1.3 = <0.05]), the % of DEG relative to all genes present in the pathway, and the overall fold change vs. -144 (green color in bars denote proportion of down-regulated DEG and red color in bars denote proportion of up-regulated DEG).

Results using IPA analysis (Fig 2) revealed a large induction of pathways associated with the inflammatory response such as ‘IL-10 Signaling’, ‘IL-6 Signaling’ and ‘Acute Phase Response Signaling’ with an important role played by the hepatic stellate cells at 12 h post-IMI via an induction of ‘Hepatic Fibrosis/Hepatic Stellate Cell Activation’ pathway. With regard to DEG associated with ‘Hepatic Fibrosis/Hepatic Stellate Cell Activation’, the DEG overlapped with other inflammatory pathways such as ‘IL-10 Signaling’ and ‘IL-6 Signaling’ and genes associated with liver tissue damage were not altered at this time. Among other noteworthy pathways highly enriched and induced were ‘Glucocorticoid Receptor Signaling’ and ‘Death Receptor Signaling’. During the inflammatory response, the liver synthesizes acute phase proteins (e.g. serum amyloid A and haptoglobin) that are associated with restoring homeostasis and providing host protection from invading microorganisms *via* inhibiting growth of bacteria. The acute phase proteins are considered a negative feedback inhibitor for the immune response [6]. Synthesis of acute phase proteins are stimulated by several cytokines, including IL-6 [35], and may partly explain the enrichment of DEG associated with ‘IL-6 Signaling’. Glucocorticoids have been well documented as a natural immunosuppressor around parturition [36] and during the inflammatory response [36]. Interleukin-10 aids in controlling the pro-inflammatory response via its anti-inflammatory properties [37]. The enrichment of both ‘IL-10 Signaling’ and ‘Glucocorticoid Receptor Signaling’ may help control the pro-inflammatory response thereby reducing risk of host tissue damage during inflammation.

Among the metabolic-related pathways, the only pathway highly enriched and induced was ‘PPAR Signaling’. The Peroxisome Proliferator-activated Receptor (**PPAR**) are ligand-dependent nuclear receptors that can control a large number of functions, but particularly control lipid metabolism [38]. The increase activation of PPARα, the most abundant PPAR isotypes in liver, during the early response to IMI challenge is somewhat expected due to the anti-inflammatory role of PPAR via increased catabolism of arachidonic acid and modulation of liver acute phase response by transrepression of inflammatory transcription factors [39].

**Fig 3:**
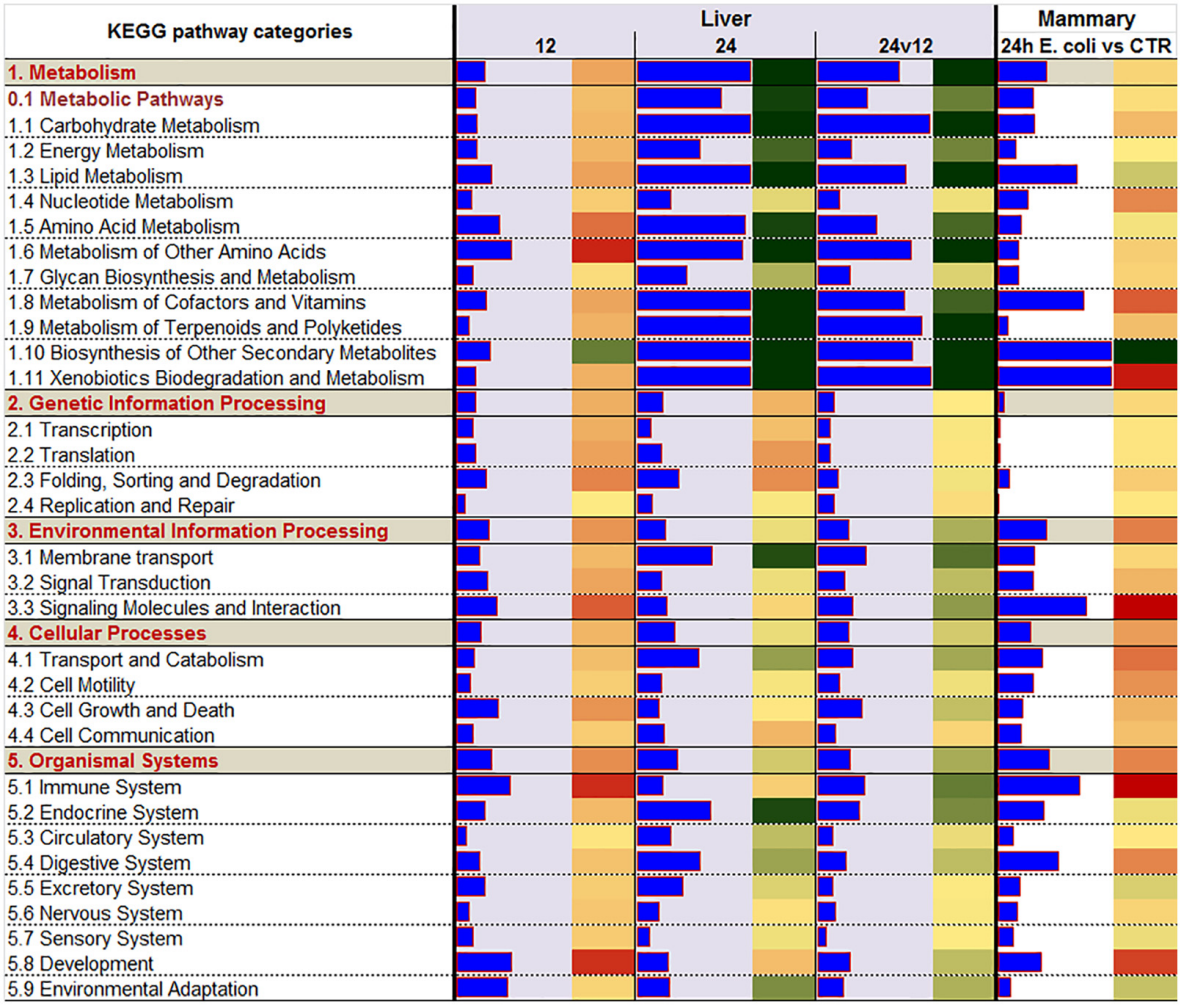
Summary of KEGG pathway results using the Dynamic Impact Approach. Impact and direction of the impact of the differentially expressed genes in bovine liver at 12 and 24 vs. -144 h relative to intramammary infection challenge with *E*. *coli* and in the infected *vs*. non-infected mammary quarters in each of the 5 main categories of KEGG pathways (in grey background and bolded-red letters) and sub-categories of pathways. Blue bars denote the impact (larger the bar, larger the impact) and the direction of the impact is denoted by the red (activated) and green (inhibited) rectangle beside each bar.

The DIA analysis (Fig 3 and S6 Fig) uncovered as the most impacted and induced pathways at 12 h post-challenge the ones associated with the ‘Immune System’ as well as metabolic-related pathways. Among immune-related pathways the ‘NOD-like receptor signaling’ and the ‘Toll-like receptor **(TLR**) signaling’ pathways were among the most impacted and induced (S6 Fig). Among metabolic-related pathways, DIA indicated a high impact and induction of pathways related to amino acid metabolism (Fig 3). There was a general induction of almost all the pathways related to amino acid metabolism but the largest induction was detected for the ‘Taurine and hypotaurine metabolism’ pathway (S6 Fig). The large effect of amino acid metabolism during inflammation is likely determined by the pro-inflammatory cytokines and appears to be an important part of the inflammatory pattern in most species [40]. The DIA analysis uncovered a relatively high impact of IMI on lipid-related pathways with ‘Fatty acid biosynthesis’, ‘Primary bile acid biosynthesis’, and ‘Steroid biosynthesis’ pathways among the most induced (S6 Fig). The larger induction of fatty acid synthesis might be associated with the increase in triglycerides accumulation in liver during IMI, at the least as observed by using LPS in cows [41]. The summary of the pathways (Fig 3) indicated as highly impacted by the IMI also pathways related to signaling. The most induced pathways related to signaling were ‘Cytokine-cytokine receptor interaction’ and ‘Chemokine signaling’ (S6 Fig). The “Folding, sorting and degradation’ category of pathways was induced by IMI (Fig 3) especially due to an induction of the ‘Protein processing in endoplasmic reticulum’ pathway (S6 Fig) indicating an induction of synthesis of secreted proteins which may be associated with the synthesis of acute phase proteins, as supported by an induction of ‘Acute Phase Response Signaling’ in IPA (Fig 2) and by increase in acute phase proteins after IMI [13]. Finally an induction of the apoptosis was also indicated by the DIA analysis (Fig 3 and S6 Fig). An increase in apoptosis of liver during inflammation has been observed previously in mice [42].

#### Hepatic transcriptomic response of liver at 24 h post-IMI

According to IPA analysis, DEG 24 h post-challenge associated with metabolism were down-regulated in liver tissue with no pathways associated with the inflammatory response significantly altered, with the exception of ‘Acute Phase Response Signaling’, which was overall induced (Fig 2). The most significantly enriched pathway at 24 h post-IMI was the ‘LPS/IL-1 Mediated Inhibition of Retinoid X Receptor (**RXR**) Function’ indicating a direct effect of the Gram-negative bacteria on inhibiting the liver metabolism through the RXR, a nuclear receptor essential for the formation of the functional heterodimer with most ligand-dependent nuclear receptors [43]. In addition, ‘Glutathione-mediated Detoxification’ is a common pathway utilized by the liver for detoxification but requires acetyl-CoA as a substrate and acetyl-CoA availability may have been low due to the inhibition of hepatic fatty acid oxidation/metabolism (Figs 2 and 3) and carbohydrate metabolism (Fig 3) during the inflammatory response.

The DIA analysis confirmed IPA results with metabolic-related pathways being the most strongly impacted and inhibited and small or no effects were detected for immune-related pathways (Fig 3). Among the most inhibited metabolic-related pathways were ‘Carbohydrate Metabolism’ (e.g., ‘Ascorbate and aldarate metabolism’, ‘Pentose and glucuronate interconversions’, ‘Glycolysis / Gluconeogenesis’, ‘Propanoate metabolism’), ‘Lipid Metabolism’ (e.g., ‘Steroid hormone biosynthesis’, ‘Primary bile acid biosynthesis’, and ‘Fatty acid metabolism’), metabolism of amino acids (e.g., ‘Valine, leucine and isoleucine degradation’, ‘Tryptophan metabolism’, ‘Glycine, serine and threonine metabolism’), metabolism of vitamins (e.g., ‘Retinol metabolism’), biosynthesis of secondary metabolites (e.g., ‘Caffeine metabolism’), and metabolism of xenobiotics (e.g., ‘Drug metabolism—cytochrome P450’) (Fig 3; S6 Fig). These results indicate that the overall metabolism in the liver was activated during early inflammation but strongly inhibited and may have been compromised in relatively later stage of inflammation. The data indicated that especially inhibited at 24 h post-IMI was the ability to oxidize fatty acids, synthesize ketone bodies, produce cholesterol, performed gluconeogenesis, and metabolize carbohydrates and amino acids. Coupled with the increase in ‘Fatty acid biosynthesis’ pathway, it is likely that the liver of the cows at 24 h post-IMI had an increase in triglycerides accumulation. This is consistent with previous observations [44,45]. Furthermore, the inhibition of hepatic gluconeogenesis by inflammation has been observed in lactating dairy cows [30]. The decrease in BHBA observed in blood is explained by a combination of increased BHBA secretion in milk [15] and a down-regulation of genes associated with hepatic ketogenesis (Fig 2). An impairment of the hepatic metabolic response during inflammation is further supported by the comparison of hepatic transcriptomic expression between 24 and 12 h post-IMI challenge (Fig 3). The DIA results indicated that DEG associated with metabolism such as ‘Carbohydrate Metabolism’, ‘Lipid Metabolism’ and ‘Metabolism of Other Amino Acids’ were the most inhibited pathways during the inflammatory response. These results provide evidence that hepatic metabolic function is compromised during inflammation and suggest that cows experiencing mastitis may be at risk for development of subsequent metabolic diseases, especially during early lactation when risk of metabolic disease is high [46].

### Up-stream Regulators Controlling the Liver Transcriptomic Response to IMI

**Fig 4:**
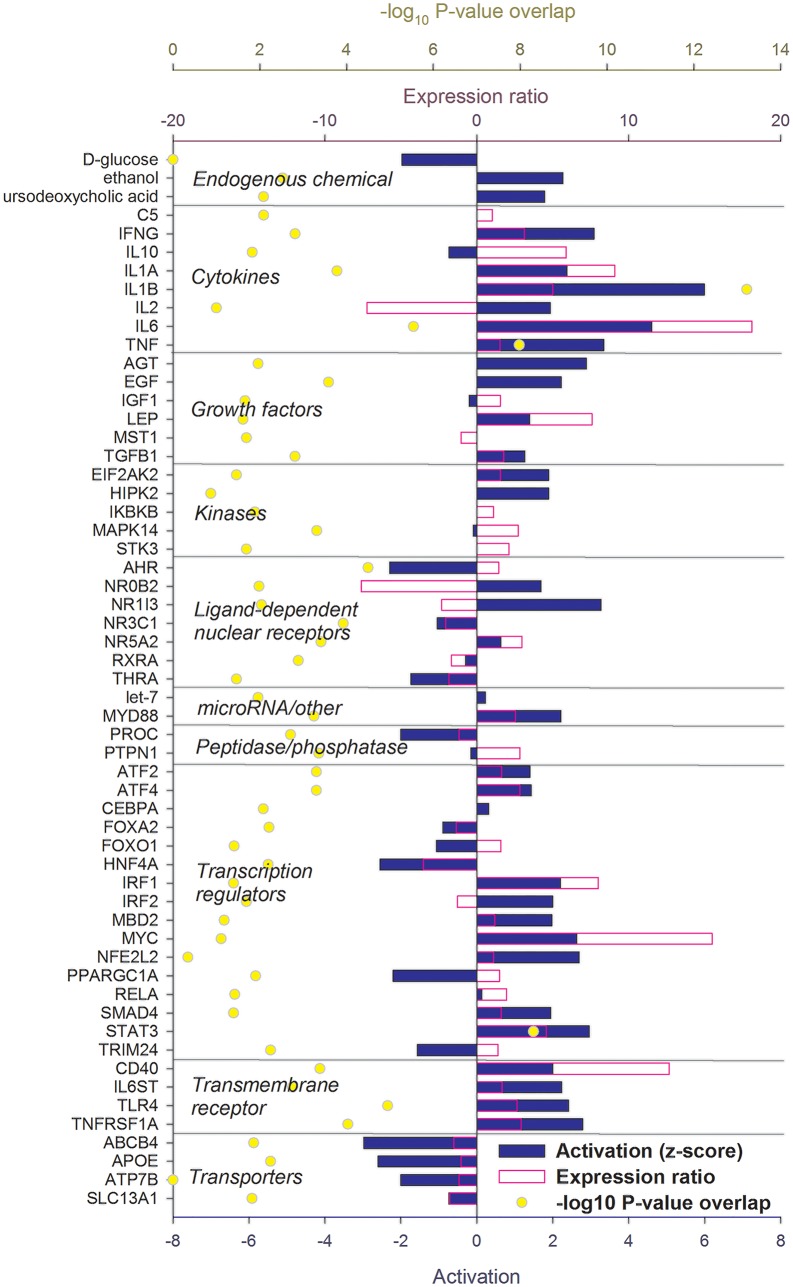
Up-stream regulators of the liver response at 12 h post-intramammary infection challenge with *E*. *coli*. Ingenuity Pathway Analysis predicts causal effects among up-stream regulators and targets (i.e., differentially expressed genes). The analysis provides the more plausible prediction of the status of the up-stream regulators (i.e. activated or inhibited) by computing an overlapping *P*-value and a Z-score. The up-stream regulators are grouped by functional categories with an activation Z-score ≥ 2 (positive = activated; negative = inhibited), significance of overlap (or enrichment; as–log_10_ of the *P*-value), and, when available and significant, the expression ratio.

**Fig 5:**
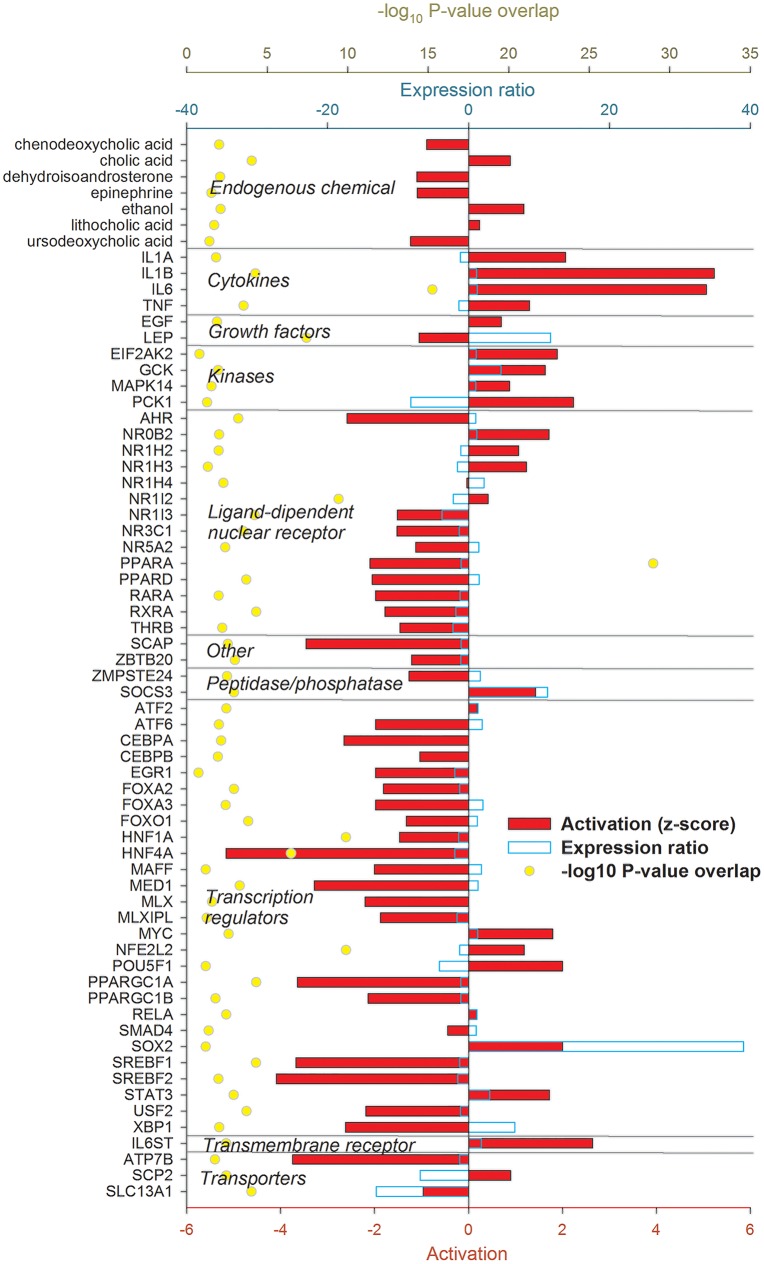
Up-stream regulators of the liver response at 24 h post-intramammary infection challenge with *E*. *coli*. Ingenuity Pathway Analysis predicts causal effects among up-stream regulators and targets (i.e. differentially expressed genes). The analysis provides the more plausible prediction of the status of the up-stream regulator (i.e. activated or inhibited) by computing an overlapping *P*-value and a Z-score. The up-stream regulators are grouped by functional categories with an activation Z-score ≥ 2 (positive = activated; negative = inhibited), significance of overlap (or enrichment; as–log_10_ of the *P*-value), and, when available and significant, the expression ratio.

The IPA analysis uncovered a large number of up-stream regulators potentially controlling the transcriptomic adaptation of the liver at 12 (Fig 4) and 24 h (Fig 5) post-IMI.

#### Hepatic response 12 h post-IMI

Genes coding for pro-inflammatory cytokines such as tumor necrosis factor alpha (*TNF*), interleukin (IL) 1α (*IL1A*) and β (*IL1B*), and IL-6 (*IL6*) were estimated to play a major up-stream controlling role. Few anti-inflammatory cytokines were also uncovered to play a regulatory role, such as *IL2* and *IL10*. Several growth factors were estimated to be important up-stream regulators such as leptin (*LEP*) and epidermal growth factor (*EGF*). Among a large number of transcription regulators (**TR**), the highest activation was estimated for *IRF1* (interferon regulatory factor 1), *MYC* (v-myc avian myelocytomatosis viral oncogene homolog), *NFE2L2* (Nuclear Factor, Erythroid 2-Like 2), *STAT3* (signal transducer and activator of transcription 3), and *SMAD4* (mothers against decapentaplegic homolog 4). The analysis by IPA indicated that *HNF4A* (hepatocyte nuclear factor 4, alpha), master regulator of lipid metabolism [47], *FOXO1* (forkhead box protein O1), essential for the regulation of gluconeogenesis [48], and *PPARGC1A* (PPAR gamma, coactivator 1 alpha), the essential co-factor for the activity of PPAR, were inhibited by the IMI. Few ligand-dependent nuclear receptors (LdNR) were deemed to play a role, among these the *NR1I3* (nuclear receptor subfamily 1, group I, member 3) and *NR0B2* (nuclear receptor subfamily 0, group B, member 2) were the most activated while *AHR* (aryl hydrocarbon receptor) and *THRA* (thyroid hormone receptor, alpha) were strongly inhibited. Several transmembrane receptors involved in the immune response were strongly induced while several membrane transporters, especially the one involved in cholesterol transport (e.g., *ABCB4* and *APOE*) were inhibited (Fig 4). Inflammation is known to negatively affect cholesterol transport in monogastrics [49] and decrease as consequence of inflammation in dairy cows [50]. The decrease in cholesterol transport was also supported in our study by the overall decrease in cholesterol following IMI (S4 Fig).

#### Hepatic response 24 h post-IMI

The pro-inflammatory cytokines regulating the transcriptomics adaptation at 12 h post-IMI were still among the main up-stream regulators also at 24 h post-IMI with a relatively strong activation. Among growth factors, *EGF* remained activated but *LEP* was inhibited at 24 h post-IMI. A large number of LdNR and TR were uncovered to play a major regulatory role by IPA. With few exceptions, all the LdNR and the TR were inhibited at 24 h post-IMI. Among these, *AHR*, *PPARA* (PPARα), *PPARD* (PPARβ/δ), *RARA* (retinoic acid receptor α) and *RXRA* (RXRα) were the most inhibited LdNR while *HNF4A*, *MED1* (mediator complex subunit 1), *PPARGC1A*, and the two SREBP (sterol regulatory element-binding protein) isoforms (i.e. *SREBF1* and *SREBF2*) were the most inhibited LR **(**Fig 5). Noteworthy is also the estimated inhibition of SCAP (SREBF cleavage activating protein), *MLX*, and *XBP1* (X-box binding protein 1). Most of the LdNR inhibited by the IMI at 24 h are involved in lipid and glucose metabolism [51,52].

Overall, the data indicated a large role of cytokines and TF in the transcriptomic regulation in the early phase of IMI but a very large importance of LdNR and TR in the transcriptomic adaptation of the liver at 24 h post-IMI, especially in controlling hepatic lipid and glucose metabolism.

### Transcription Network Controlling the Transcriptomic Adaptation in Liver after IMI

**Fig 6:**
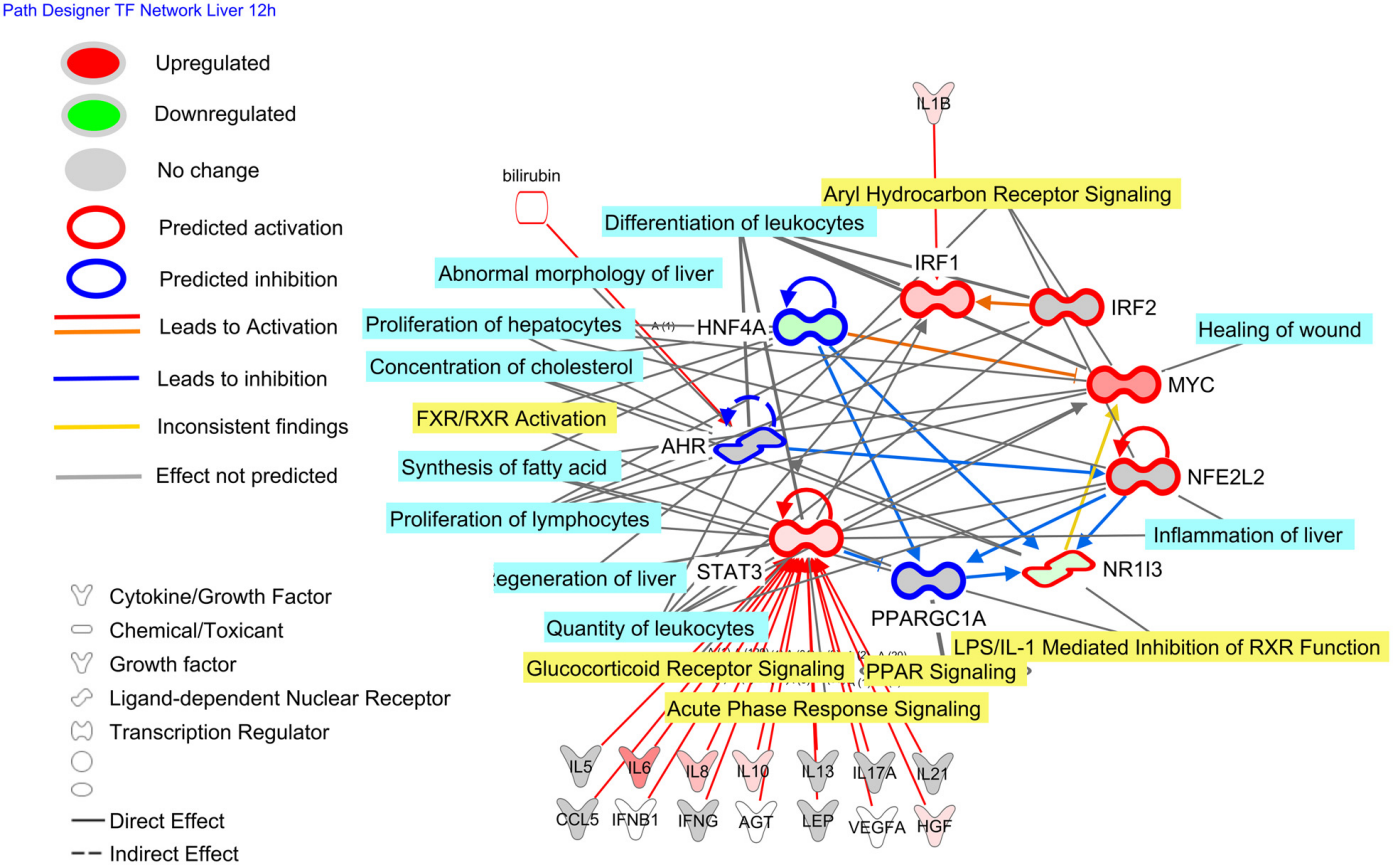
Transcriptional factor network of the liver response at 12 h post-intramammary infection challenge with *E*. *coli*. Reported is the cross-talk among the transcription factors (**TF**) in bovine liver uncovered to be either activated or inhibited by Ingenuity Pathway Analysis (**IPA**; from Fig 3). The potential up-stream endogenous chemicals, growth factors, and cytokines affecting the activation or inhibition of the TF are estimated by IPA. The TF network included all the TF and ligand-dependent nuclear receptors. The network allows visualizing the interaction among TF, the overall estimated activation or inhibition, the up- or down-regulation, the up-stream activators or inhibitors, and the enriched functions (light blue shade) or pathways (yellow shade) with the larger number of differentially expressed genes.

**Fig 7:**
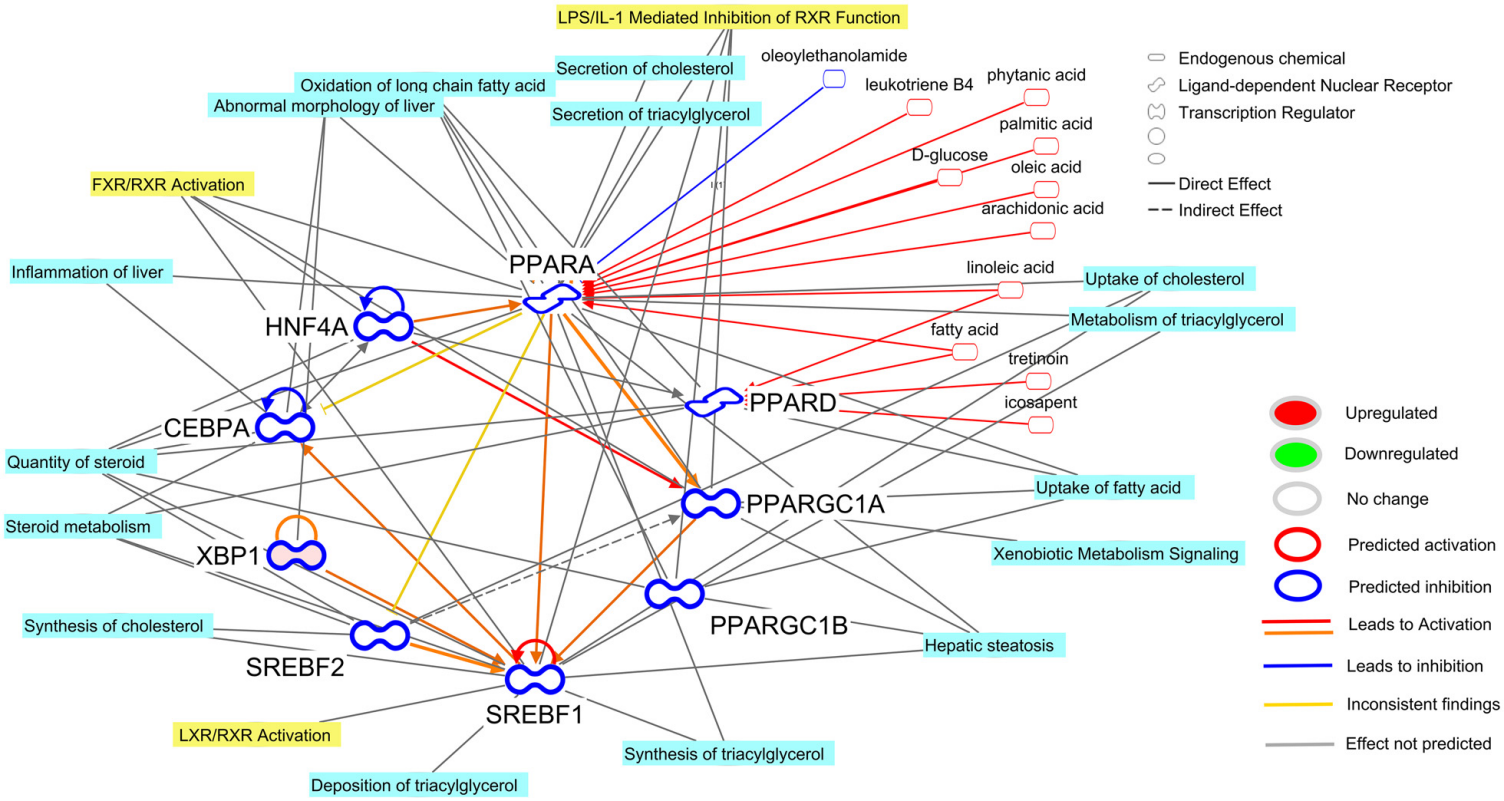
Transcriptional factor network of the liver response at 24 h post-intramammary infection challenge with *E*. *coli*. Reported is the cross-talk among the transcription factors (TF) in bovine liver uncovered to be either activated or inhibited by Ingenuity Pathway Analysis (IPA). Using IPA database, it revealed the potential up-stream endogenous chemicals, growth factors, and cytokines affecting the activation or inhibition of the TF. The TF network included all the transcription factors and ligand-dependent nuclear receptors. The network allows visualizing the interaction among TF, the overall estimated activation or inhibition, the up- or down-regulation, the up-stream activators or inhibitors, and the enriched functions (light blue shade) or pathways (yellow shade) with the larger number of differentially expressed genes.

The analysis of the transcriptional network among the up-stream regulators deemed to have played a major role in the transcriptomics adaptation of the liver to IMI (Figs 4 and 5), uncovered a relative small number of TF with potential large cross-talk capabilities (Figs 6 and 7). At 12 h post-IMI, the network analysis revealed a tight network among several predicted activated TR such as *MYC*, *STAT3* and *IRF1*, and several predicted inhibited LdNR and TR, such as *NR1I3*, *AHR*, *HNF4A* and *PPARGC1A* (Fig 6). The *STAT3* was predicted to be activated by a plethora of cytokines and growth factors with *IRF1* activated by IL1-β (Fig 6). This prediction is indicative of *STAT3* being a central hub in the response to liver inflammation with the activation of *MYC*, *NR1I3*, and *NFE2L2* at 12 h post-IMI mainly due to the activation of STAT3 by cytokines. However, the network also indicated that the inhibited LdNR and TR had a negative regulatory role toward several of the pro-inflammatory TR (Fig 6). Thus, the activation of the networks toward a pro-inflammatory response could have been initially be driven by cytokines mainly through STAT3 but then the inhibition of several TR and LdNR had reduced the inhibitory role, amplifying the pro-inflammatory response. The importance of the TR and LdNR of the network is highlighted also by their significant association with several pathways and functions with an important role in the early response to inflammation, such as ‘Glucocorticoid Receptor Signaling’, ‘Acute Phase Response Signaling’, ‘Regeneration of Liver’, ‘Inflammation of Liver’, ‘Proliferation of Lymphocytes’ and ‘Synthesis of Fatty Acid’.

At 24 h post-IMI, the transcription network was composed exclusively by metabolic-associated LdNR and TF including *PPARA*, *PPARD*, SREBP isoforms, and *HNF4A* (Fig 7). Among these TF the PPAR are known to be highly regulated by endogenous or dietary compounds, such as fatty acids, leukotriene B4, and glucose [38]. However, according to the predicted up-stream regulators analysis, the importance of endogenous chemicals is not as large as cytokines (Fig 5). It is known that several of the LdNR and especially PPAR isotypes are inhibited indirectly by cytokines [53] which, however, was not predicted to have played a prominent role at 24 or 12 h post-IMI. The transcriptional network at 24 h post-IMI (Fig 7) was significantly associated with lipid-related metabolism, including oxidation of fatty acids and synthesis of cholesterol and triglycerides which were all deemed to be inhibited at 24 h post-IMI. The above results are indicative of a prominent role of metabolic-related LR and LdNR on the coordination of the metabolic shut down of the liver at 24 h post-IMI.

### Functional Analysis of Mammary Transcriptome: Large Induction of Inflammatory Signaling and Reduced Milk Fat Synthesis

Differentially expressed genes up-regulated in mammary tissue 24 h post-IMI challenge were associated with the inflammatory response and down-regulated DEG were associated with lipid metabolism. The DIA analysis (Fig 3) revealed that the most impacted pathways primarily activated in mammary tissue 24 h post-IMI challenge were associated with the ‘Immune System’, ‘Signaling Molecules and Interaction’ (i.e., ‘Jak-STAT signaling pathway’), and ‘Xenobiotics Biodegradation and Metabolism’. Only two categories of pathways were predicted to be primarily inhibited in mammary tissue after IMI challenge, i.e. the ‘Biosynthesis of Other Secondary Metabolites’ and ‘Lipid metabolism’. Surprisingly, no pathways or functions relating to the utilization of BHBA in the mammary transcriptome were observed and is partly supported by the down-regulation in expression of 3-hydroxybutyrate dehydrogenase isoforms (*File S1*), genes coding for a key proteins for the utilization of 3-hydroxybutyrate. Increased transfer of BHBA from circulation to the mammary gland partly explains the decrease in blood BHBA relative to increases in milk BHBA observed in this study [15]. The mammary transcriptome data indicate little or no utilization of BHBA by mammary tissue. The role, if any, of BHBA in milk during IMI is unclear and warrants further investigation. The importance of lipid metabolism was highlighted by ‘Fatty acid biosynthesis’ and ‘Glycerolipid metabolism’ being among the top impacted and inhibited pathways (S6 Fig). Among the many immune-related pathways that were highly impacted and activated, the ‘Chemokine signaling pathway’ and the ‘NOD-like receptor signaling’ were the most important (Fig 3 and S6 Fig).

**Fig 8:**
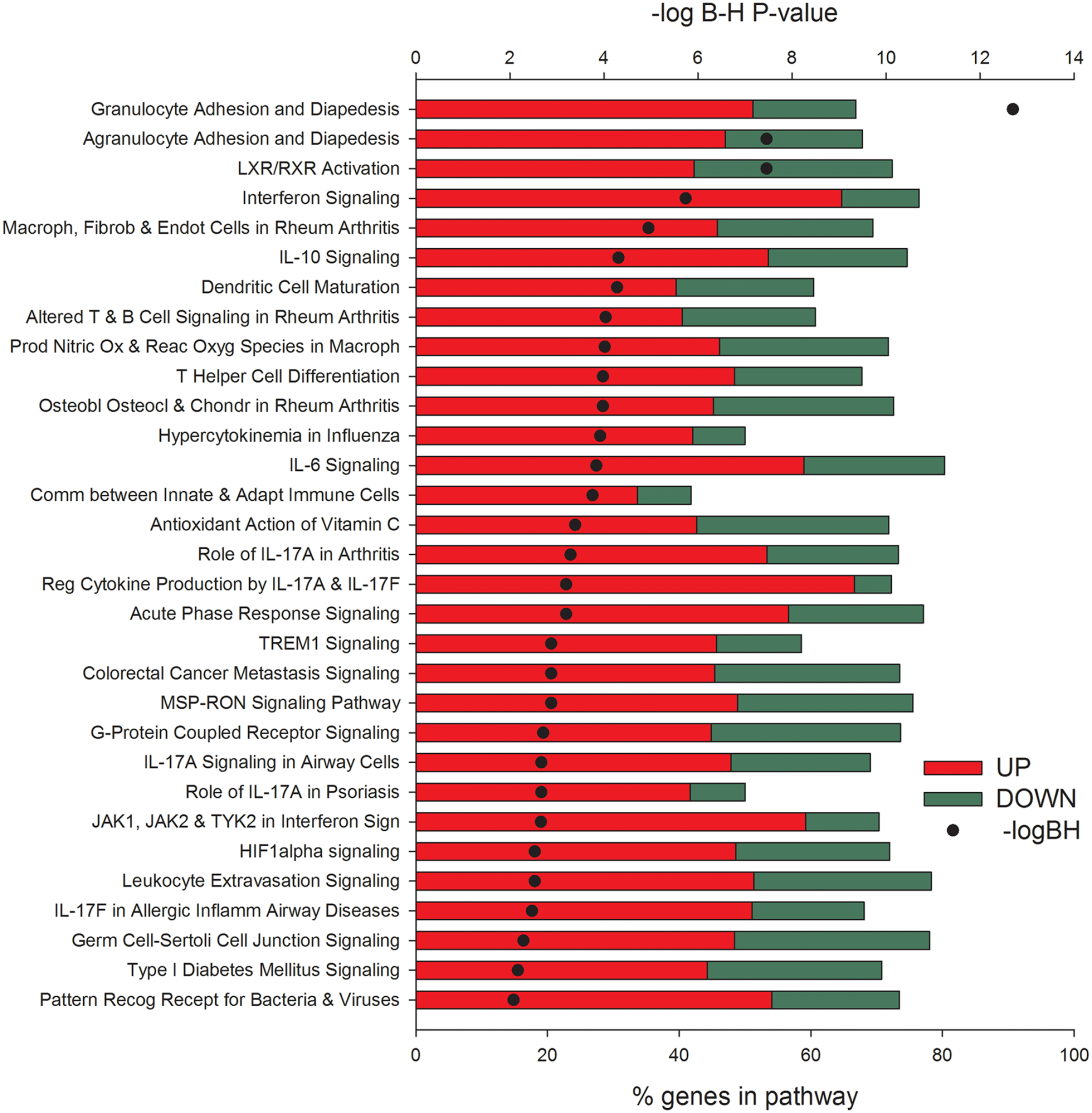
Most enriched pathways in infected vs. non-infected mammary quarters at 24 h after intramammary infection challenge with *E*. *coli*. Reported are the significance of enrichment (as–log_10_ of the Hochberg-Benjamini false discovery rate [-logBH; >1.3 = <0.05]), the % of differentially expressed genes relative to all genes present in the pathway, and the overall expression ratio of infected vs. non-infected (green bars denote down-regulation and red bars up-regulation) uncovered by Ingenuity Pathway Analysis.

The results from the IPA analysis (Fig 8) supported DIA results. All the most enriched pathways were associated with the activation of the inflammatory response such as adhesion and diapedesis of leukocytes, ‘Interferon Signaling’, IL-10 and IL-6 Signaling, and ‘Acute Phase Response Signaling’. The ‘LXR/RXR Activation’ was the only metabolic-related pathway significantly enriched (Fig 8). Despite having the majority of DEG associated with these pathways up-regulated by IMI, several of the DEG were strongly down-regulated and are known to be associated with milk fat synthesis. Among these are *LPL* (lipoprotein lipase), *FASN* (fatty acid synthase), *SREBF1*, and *ACACA* (acetyl-CoA carboxylase alpha) (S5 Fig). No down-regulation of *PPARG* (PPARγ) or *NR1H3* (LXR) were detected (S5 Fig) despite the fact that milk fat synthesis was substantially reduced post-IMI [15]. Previous data with IMI using *Streptococcus uberis* were indicative of PPARγ being important in the observed reduction of milk fat synthesis [54]. These data with other evidences allowed proposing a role of PPARγ in the control of milk fat synthesis in the bovine mammary gland [38]. The data from the present paper only partly support a role of PPAR and LXR being important in the decreased milk fat synthesis due to *E*. *coli* IMI, mostly inferred by the decrease in expression of target genes; however, none of the bioinformatics analysis results were indicative of these two LdNR being crucial in the *E*. *coli* IMI response.

**Fig 9:**
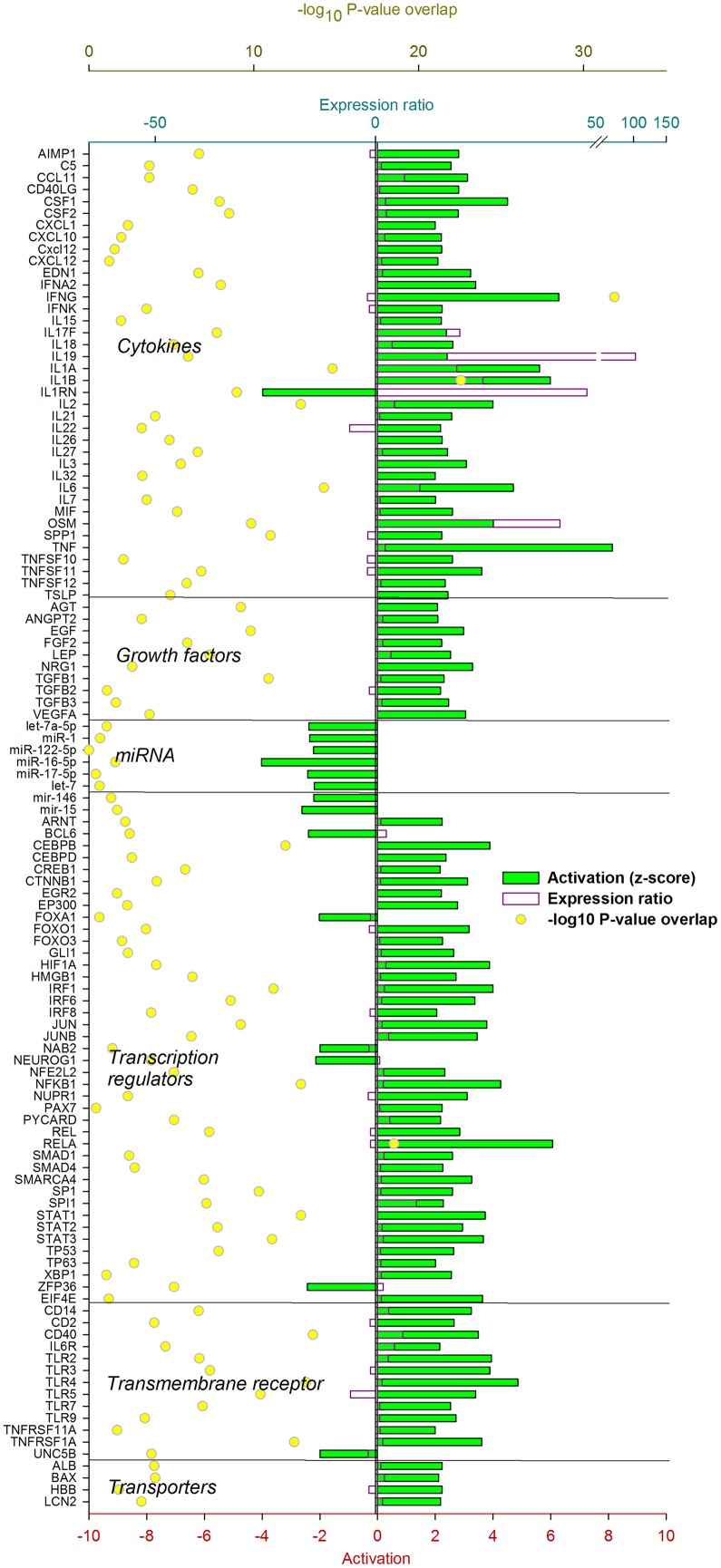
Up-stream regulators of the mammary response at 24 h after intramammary infection challenge with *E*. *coli*. Ingenuity Pathway Analysis predicts causal effects among up-stream regulators and targets (i.e. differentially expressed genes; DEG). The analysis provides the more plausible prediction of the status of the up-stream regulator (i.e. activated or inhibited) by computing an overlapping *P*-value and a Z-score. The up-stream regulators are grouped by functional categories with an activation Z-score (positive = activated; negative = inhibited), significance of overlap (or enrichment; as–log_10_ of the *P*-value), and, when available and significant, the expression ratio.

Overall, the results from DIA and IPA clearly are indicative of a marked activation of pathways and functions associated with the immune response and inhibition of pathways associated with lipid synthesis in mammary tissue 24 h post-IMI challenge with *E*. *coli*. The analysis of up-stream regulators (Fig 9) revealed a primary role of a large number of cytokines, growth factors, and TR, with almost all predicted to be highly activated in the coordination of the transcriptomic adaptation to IMI by the mammary tissue. Among the up-stream regulators, the largest predicted activation was uncovered for *INFG* (interferon gamma) and *TNFA* among DEG coding for cytokines, whereas *EGF*, *NRG1* (neuregulin 1; that can bind EGF receptor), and *VEGF* (vascular endothelial growth factor) among the growth factors, *RELA* (v-rel avian reticuloendotheliosis viral oncogene homolog A) and *NFKB1* (nuclear factor of kappa light polypeptide gene enhancer in B-cells) were among the TR most highly activated in mammary tissue at 24 h post-IMI. The data depict a large importance of TR involved in inflammation and immune response with an interesting induction of epithelial proliferation (i.e., *EGF*) [55] and vasculogenesis (i.e., *VEGF*). Several transmembrane receptors were predicted to be highly activated, among these prevailed several of the TLR which are involved in the inflammatory response (Fig 9). Contrary to the liver, where no miRNA (microRNA) were predicted to be up-stream regulators, the mammary tissue response resulted in a relatively large number of miRNA inhibited with miR16-5p be the most inhibited (Fig 9). Interestingly, when compared with the actual data (S5 Fig), none of the predicted down-regulated miRNA by IPA were actually affected by the IMI. Up to 11 miRNA were significantly affected by the IMI (FDR<0.001) with 9 of them up-regulated and 2 down-regulated (miR30F and miR33b). Among the up-regulated miRNA, the miR23A was induced >420-fold in mammary 24 h post-IMI (S5 Fig). The up-regulation of miR23A can be associated with the activation of TLR, especially TLR2 [56]. Whether the miRNA expression observed serves as a feedback signal altering the mammary transcriptome is unclear.

**Fig 10:**
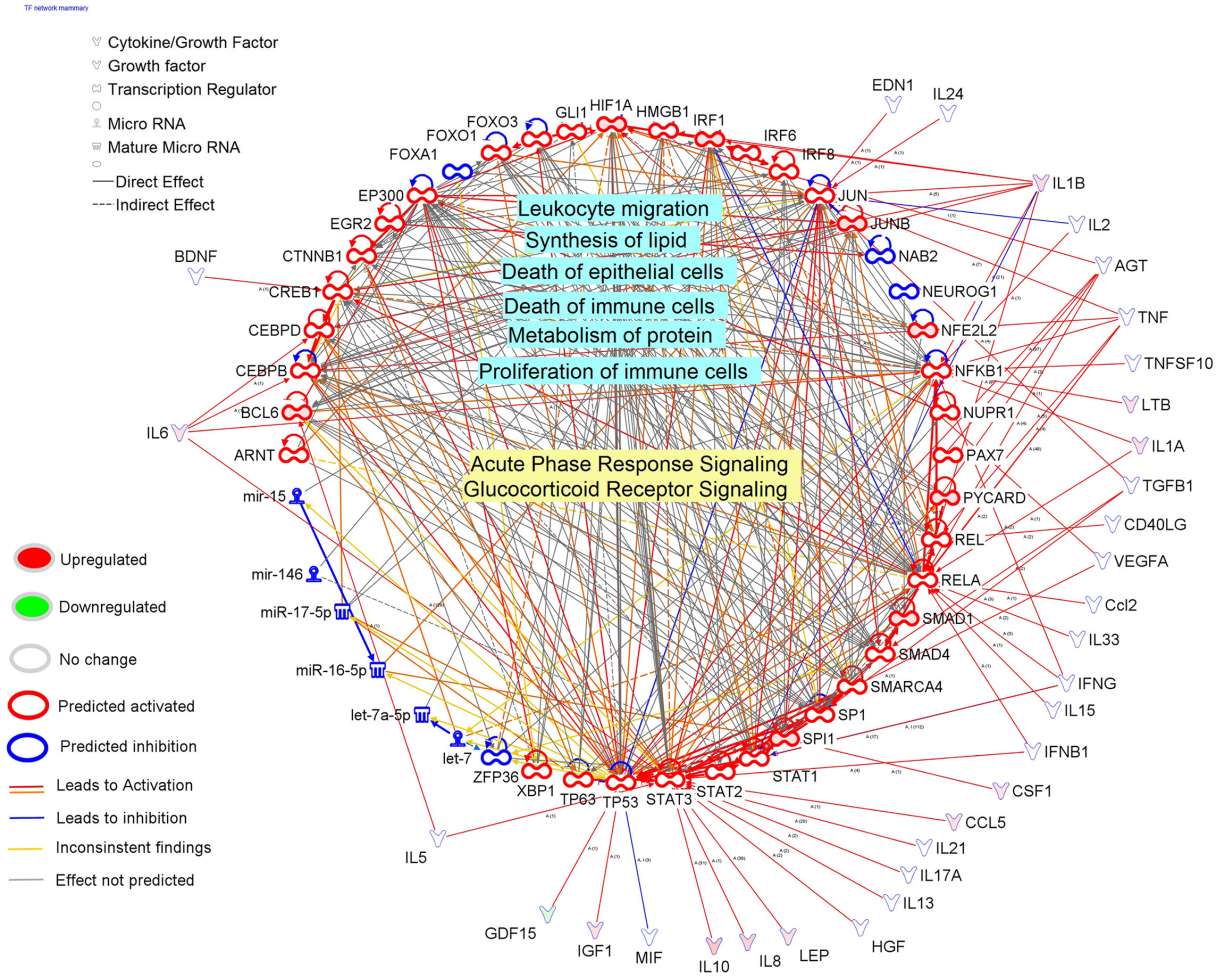
Transcriptional factor network of the mammary tissue response at 24 h after intramammary infection challenge with *E*. *coli*. Reported is the cross-talk among the transcription factors (TF) in bovine mammary tissue uncovered to be either activated or inhibited by Ingenuity Pathway Analysis (IPA). The IPA database uncovered the potential up-stream endogenous chemicals, growth factors, and cytokines affecting the activation or inhibition of the TF. The TF network included all the transcription factors and ligand-dependent nuclear receptors. The network allows visualizing the interaction among TF, the overall estimated activation or inhibition, the up- or down-regulation, the up-stream activators or inhibitors, and the enriched functions (light blue shade) or pathways (yellow shade) with the larger number of differentially expressed genes.

The transcription regulation network controlling the transcriptomic adaptation of the mammary tissue 24 h post-IMI was very large (Fig 10). Most of the TR were deemed to be activated and associated with inflammatory response under the control of a plethora of cytokines. The TR in the network were highly associated with the acute phase response and activity and proliferation of leukocytes. The TR of the network were also estimated to be associated with synthesis of lipid and metabolism of proteins (Fig 10).

Overall the data are indicative of a large inflammatory response of the mammary at 24 h post-IMI with little or no effect on metabolism, with the exception perhaps of some negative effects on milk fat synthesis supporting the decrease in milk fat synthesis observed [15]. The adaptation of mammary tissue was highly coordinated by cytokines that appeared to have driven the inflammatory signaling network of the mammary.

Both RNAseq reported here and microarray analyses in liver [7,17] and mammary tissue [13] resulted in similar conclusions. In mammary tissue, an up-regulation of DEG associated with the inflammatory response and a down-regulation of DEG associated with lipid metabolism were observed in both studies. In liver, DEG associated with the inflammatory response were up-regulated during the early inflammatory response (i.e. 12 h post-IMI challenge) whereas DEG associated with metabolism were down-regulated during peak inflammatory response (i.e. 24 h post-IMI challenge). Results indicate that during the early response of mastitis an increase in both pro-and anti-inflammatory factors may help control inflammation while minimizing damage to liver tissue. In addition, results suggest an increase in the synthesis of acute phase proteins in response to pro-inflammatory cytokines (e.g. IL-1β, IL-6 and TNF-α) released primarily from resident macrophages and mammary epithelial cells during mastitis. During peak inflammatory response (i.e. 24 h post-IMI challenge), hepatic tissue shifts from an inflammatory state to a reduction in the liver’s ability to metabolize nutrients, especially energy and protein metabolism.

### Cross-talk Between Liver and Mammary Inferred by the Transcriptomic Analysis

**Fig 11:**
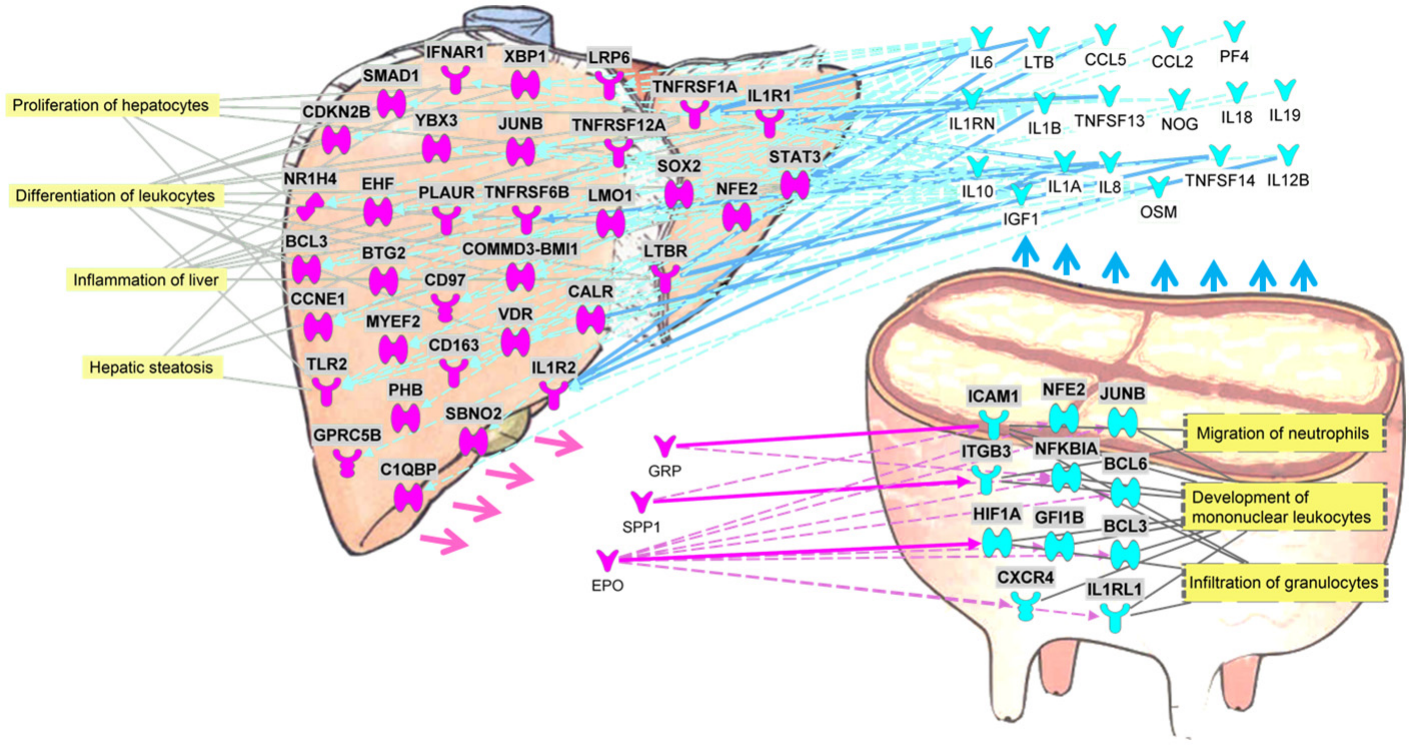
Potential cross-talk between liver and mammary at 24 h after intramammary infection challenge with *E*. *coli*. Purple objects for liver and blue for mammary denote genes with expression ≥2-fold at 24 h after intramammary infection challenge with *E*. *coli*. Differentially expressed genes that code for proteins that are either released (→) by the tissue as cytokines and growth factors (drawn outside the organs) or function as receptor (drawn inside the organ). Solid ➞ denote direct and dashed ⇢ denote indirect activation of receptors in one organ by cytokines or growth factors with a potential increased release from the other organ. In yellow shade are most enriched functions of the affected receptors.

In Fig 11 is shown the cross-talk between liver and mammary tissue as inferred by the DEG with an expression ≥2-fold at 24 h post-IMI challenge with *E*. *coli*. The use of a more stringent criteria identified approximately 2,300 DEG in liver at 24 h post-IMI compared to -144 h and 1,800 DEG in mammary tissue in the IMI vs. control (51.6% and 76.8% of the DEG compared to the functional analysis reported above).

The analysis revealed substantial cross-talk between the two tissues with a communication almost unidirectional, i.e. mammary to liver. The IPA analysis predicted that the mammary tissue primarily had a large increase in secreted inflammatory cytokines, e.g. IL-10, IL-6 and IL-1β, that interacted with liver receptors highly expressed during IMI, i.e. *TLR2*, *IL1R1*, *TNFRSF1A*, *STAT3*, and *BCL3* (B-cell CLL/lymphoma 3) with a consequent increase in hepatic proliferation and regeneration and the inflammatory response (primarily acute phase protein response; Fig 2).

The IPA analysis identified 3 up-regulated DEG in liver at 24 h post-IMI, *GRP* (gastrin-releasing peptide), *SPP1* (secreted phosphoprotein 1; osteopontin), and *EPO* (erythropoietin), that encode for signaling proteins that may potentially interact with receptors involved in the migration of neutrophils, development of mononuclear leukocytes and infiltration of granulocytes in mammary tissue (Fig 11).

The gastrin-releasing peptide was originally classified as a neurotransmitter [57] and more recently has been associated with the endocrine response primarily as a regulatory peptide in the cow reproductive tract [58]. The protein encoded by *SPP1*, i.e. osteopontin, is expressed mainly in the bone and kidney and is involved in the attachment of osteoclasts to the mineralized bone matrix. Osteopontin also acts as a cytokine that regulates the immune-mediated disease response [59]. The SPP1 protein has been associated with inflammation, metabolic diseases, fatty liver, and liver fibrogenesis in human [60,61]. The up-regulation of *SPP1* at 24 h post-IMI challenge in the liver may have played a role in the impairment of hepatic metabolic function during inflammation as well as maintenance of the inflammatory response in the mammary tissue.

## Summary and Conclusions

**Fig 12:**
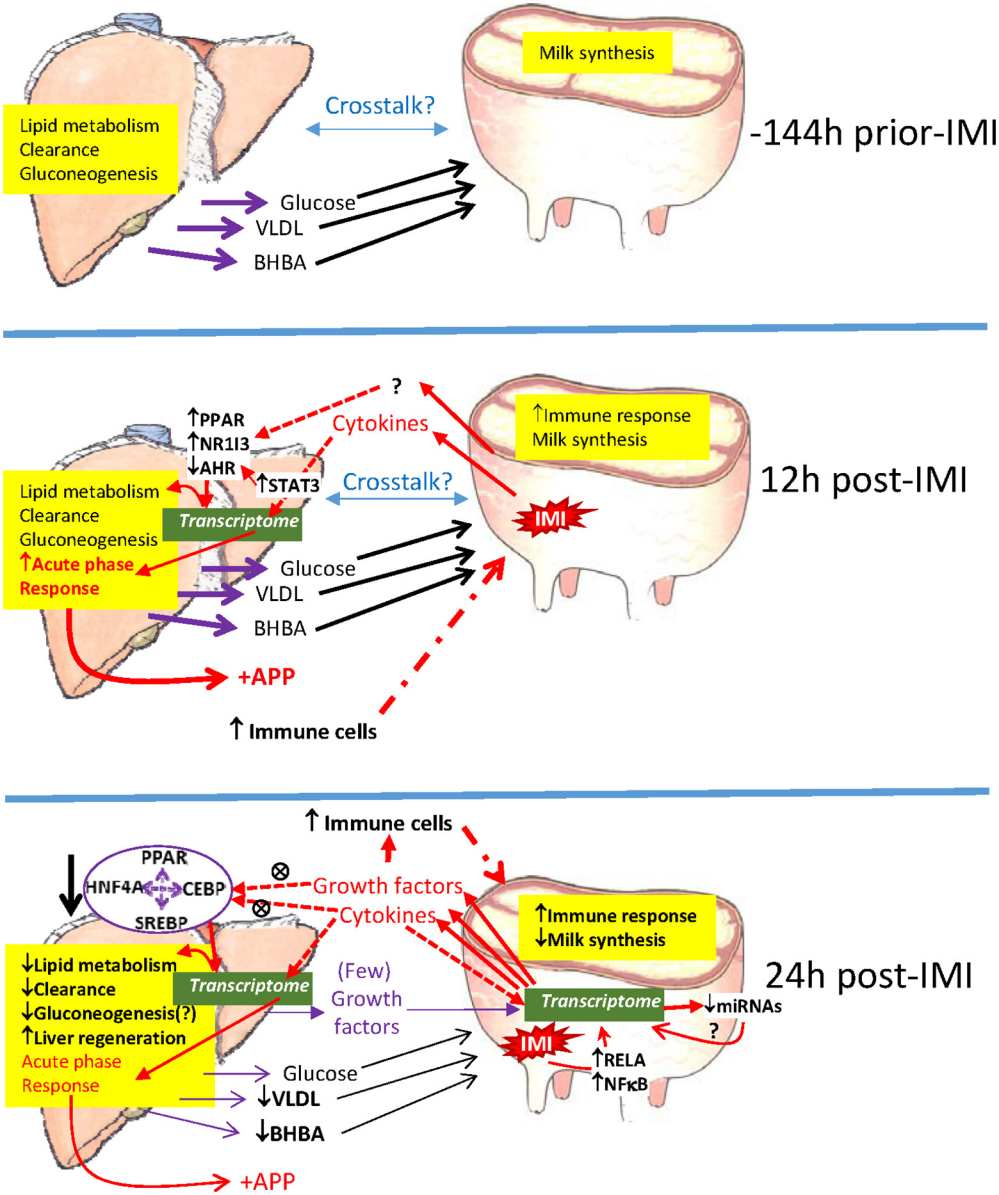
Model summarizing main findings. Based on known physiology of the dairy cows we assumed that before intramammary infection (-144) the liver was performing its usual physiological functions, such as clearance/detoxification and producing and releasing in the bloodstream glucose, VLDL, and BHBA through gluconeogenesis and lipid metabolism. The compounds released by the liver are taken up by the mammary gland to synthesize milk. Cross-talk between the two organs is plausible. In the early response to intramammary infection (i.e. 12 h), our data indicated a strong inflammatory response of the liver with increased synthesis of positive acute phase proteins (see also S4 Fig) with no effects on metabolism. The data indicated that the acute phase response was mainly driven by STAT3 with PPAR and NR1I3 apparently the more activated and AHR the more inhibited ligand-dependent transcription factors. The effect on the liver is likely derived by the pro-inflammatory cytokines released by the infected mammary quarter during the first phases of IMI. At 24h post-IMI, the cytokines and growth factors released by the mammary tissue during IMI decreased the overall metabolism of the liver, especially acting through a relatively small network of transcription factors related to metabolism. As consequence the liver may have experienced a reduce lipid and glucose metabolism and momentarily impairment of clearance or detoxification. This is also supported by the significant decrease in expression of negative acute phase proteins at this time (S4 Fig). The data indicated also an induction of liver regeneration, possibly driven by growth factors released by the infected mammary quarter. Our data indicated a very little communication form the liver to the mammary tissue which was still experiencing a large inflammatory response mainly driven by the cytokine- factors NFKB and RELA. It is also possible that observed change in expression of miRNA may have played a role in maintaining the inflammatory status in the mammary tissue. Our analyses revealed substantial cross-talk between the two tissues with a communication almost unidirectional, i.e. mammary to liver, via various cytokines and growth factors altered during inflammation. The mammary tissue appears to have synthesized positive acute phase proteins but did not experience a reduction of negative acute phase proteins (S4 Fig). By 24 h post-IMI, milk synthesis decreased in the infected mammary gland, primarily milk fat synthesis. AHR = aryl hydrocarbon receptor; APP = acute phase proteins; BHBA = beta hydroxybutyrate; CEBP = CCAAT/enhancer-binding protein alpha isoform d; HNF4A = hepatocyte nuclear factor 4, alpha; IMI = intramammary infection; NFKB = nuclear factor of kappa light polypeptide gene enhancer in B-cells; NR1I3 = nuclear receptor subfamily 1, group I, member 3; PPAR = peroxisome proliferator-activated receptors; RELA = v-rel avian reticuloendotheliosis viral oncogene homolog A; SREBP = sterol regulatory element-binding protein; STAT3 = signal transducer and activator of transcription 3; VLDL = very low density lipoproteins.

Fig 12 shows the overall project summary and conclusions. Our data indicated that the liver had strong transcriptomic response at 12 h post-IMI characterized by a large inflammatory-type activity primarily via the acute phase response with minimal changes observed regarding metabolism and clearance. At 24 h post-IMI, the transcriptomic data indicated that the liver substantially decreased the overall metabolism, but in particular lipid metabolism. This impaired metabolic response of liver at the transcription level where a down regulation of DEG associated with hepatic gluconeogenesis and ketogenesis was observed that partly explains changes in metabolites observed in blood. The analysis indicated that the transcriptomic response of the liver was mainly influenced by cytokines and growth factors but with a substantial role of ligand-dependent nuclear receptor (i.e. *PPAR*) at 24 h post-IMI. A relatively small core of such TF appeared to have a strong interaction and had a large weight regulating the hepatic response at 24 h post-IMI (e.g. PPAR isotypes and related co-activators, HNF4A, SREBP isoforms, CEBP [CCAAT/enhancer-binding protein alpha isoform d], and XBP1). Coupling the metabolic response in blood and milk with transcriptomic responses in liver, our results indicate impaired liver metabolism during later stages of inflammation.

The transcriptome analysis of the mammary tissue at 24 h post-IMI indicated a large immune response of the tissue with very little effect on metabolism, except a likely inhibition of lipid synthesis. Contrary to the liver, the transcriptomic adaptation of the mammary tissue appeared to be driven by a large network of up-stream regulators, with a large sensitivity to cytokines. The inflammatory transcriptomic response of the mammary tissue is supported by the inflammatory response observed in milk where dramatic increase in inflammatory mediators was observed after IMI with *E*. *coli*.

The analysis of cross-talk uncovered a large communication from the mammary to the liver to coordinate the inflammatory response with very few factors potentially released by the liver to control the response of the mammary tissue during IMI. Our data also indicate that the mammary tissue did not directly influence the decreased metabolism of the liver at 24 h post-IMI but, likely, indirectly impacted hepatic metabolism via stimulation of the hepatic inflammatory response.

A summary of the most relevant findings in the present experiment are reported in Fig 12. In conclusion, our data revealed a different response of the liver and mammary tissue during IMI, with a similar overall inflammatory-like response of the mammary tissue in the quarter treated with IMI vs. the control quarter at 24 h post-treatment and liver at 12 h vs. -144 h post-IMI with also a large increase in expression of positive acute phase proteins related genes (S4 Fig). Despite this, the metabolism of the mammary tissue was not significantly affected. As a result, the mammary tissue did not experience the “shutdown” of specific functions, i.e. metabolism and clearance, as observed in the liver, at 24 h post-IMI. The momentarily impairment of metabolism and clearance capability of the liver as consequence of the IMI might be partly explained by the shift in partitioning of nutrients towards the immune response. Due to the pivotal role played by the liver in controlling overall nutritional economy, metabolism, clearance from xenobiotics, and the immune response, our findings of a transcriptionally driven decline of critical functions in the liver provide further evidence of the negative consequence on overall performance of dairy cows affected by mastitis. The IMI response appeared to be highly coordinated by the potential increase in signaling between the two tissues with a strong regulatory role of the mammary tissue toward the liver via signaling molecules. Overall, the data revealed a previously unknown cross-talk between mammary and liver coordinating the response to IMI.

## Supporting Information

S1 Fig Concentrations of alkaline phosphatase (A), N-acetyl-β-D-glucosaminidase (NAGase; B) and lactate dehydrogenase (LDH; C) in milk from cows (n = 16) after intramammary challenge during early lactation. *Differences (*P* < 0.05) when compared to h = 0.
(DOCX)

S2 Fig Changes in daily feed intake (A) and milk yield (B) from cows (n = 16) after intramammary challenge during early lactation. *Differences (*P* < 0.05) when compared to h = 0.
(DOCX)

S3 Fig Plasma concentrations of non-esterified fatty acids (NEFA; A), beta-hydroxybutyrate (BHBA; B), glucose (C) and cholesterol (D) from cows (n = 16) after intramammary challenge during early lactation. *Differences (*P* < 0.05) when compared to h = 0.
(DOCX)

S4 Fig Expression of genes coding for selected pro-inflammatory cytokines and positive and negative acute phase proteins (data as also reported in S5 Fig). (JPG)

S5 Fig Complete dataset with false discovery rate (FDR) for the overall time or treatment effect, expression ratio, and *P*-value between comparisons. (XLSX)

S6 Fig Dynamic Impact Approach of the KEGG pathways analysis for both liver and mammary tissue. Presented are the summary of the categories of pathways, the details of each pathway, and sorted in descending order of impact in each comparison.
(XLSX)

S7 Fig Results of the pathway and function analysis of Ingenuity Pathways Analysis for both liver and mammary tissue. (XLSX)

S8 Fig Complete results of the predicted up-stream regulators by Ingenuity Pathway Analysis for both liver and mammary tissue. (XLSX)
