# Supplementary figures and images for: The Impact of Intramammary Escherichia coli Challenge on Liver and Mammary Transcriptome and Cross-Talk in Dairy Cows during Early Lactation Using RNAseq
Source: PLoS One. 2016 Jun 23;11(6):e0157480. doi: 10.1371/journal.pone.0157480 (PMC4919052; doi:10.1371/journal.pone.0157480)

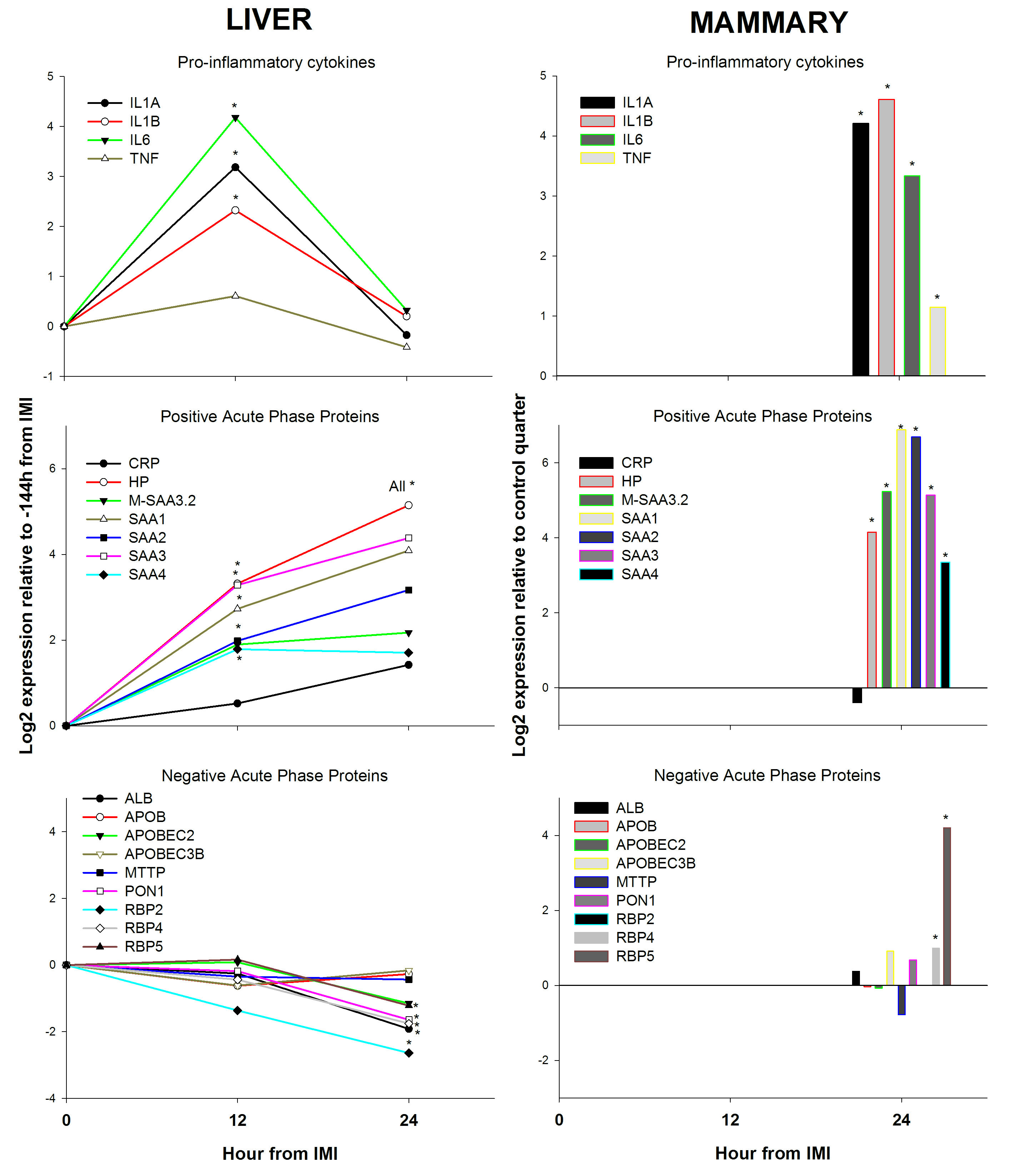

Supplement: S4 Fig — (JPG) [file pone.0157480.s004.jpg]
