# Supplementary material for: The Impact of Intramammary Escherichia coli Challenge on Liver and Mammary Transcriptome and Cross-Talk in Dairy Cows during Early Lactation Using RNAseq
Source: PLoS One. 2016 Jun 23;11(6):e0157480. doi: 10.1371/journal.pone.0157480 (PMC4919052; doi:10.1371/journal.pone.0157480)
Supplement: Supplementary file 2 [file pone.0157480.s001.docx]

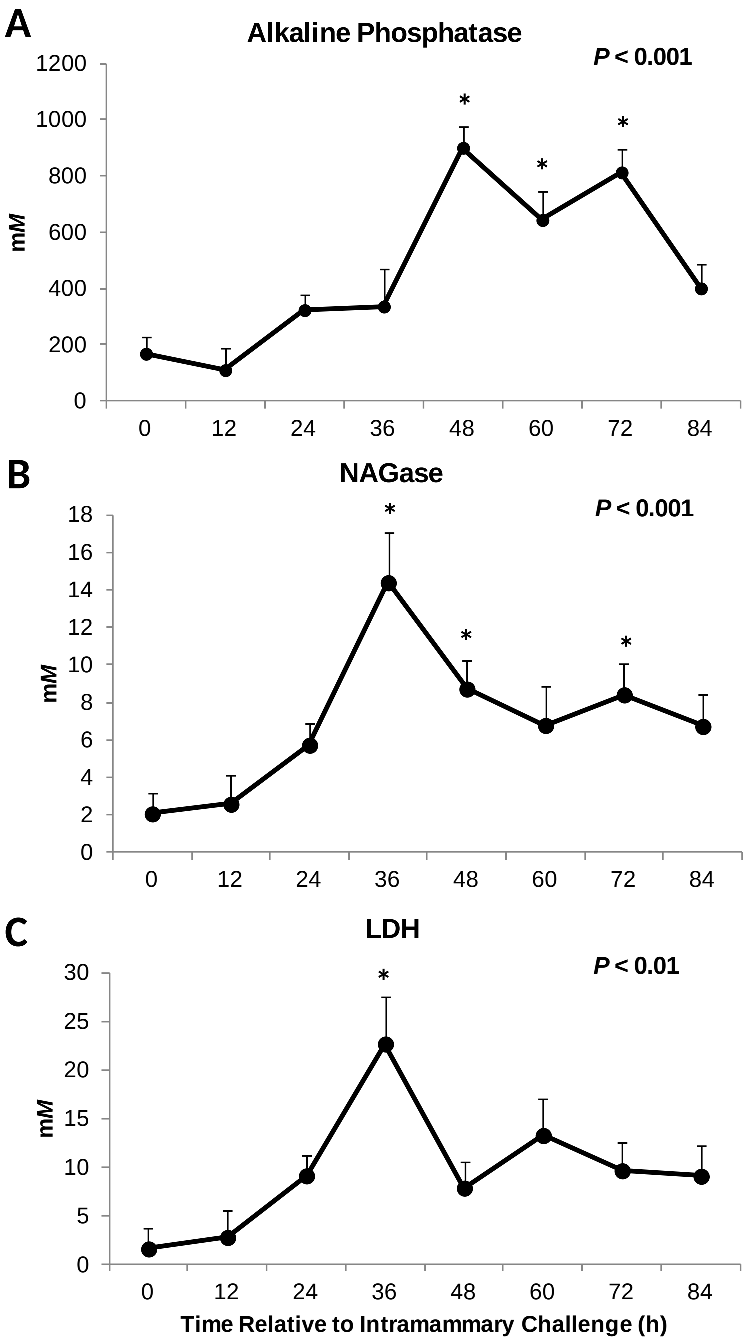

A
Alkaline Phosphatase
P < 0.001
mM
1200
1000
800
600
400
200
0
0
12
24
36
48
60
72
84
B
NAGase
P < 0.001
mM
18
16
14
12
10
8
6
4
2
0
0
12
24
36
48
60
72
84
C
LDH
P < 0.01
mM
30
25
20
15
10
5
0
0
12
24
36
48
60
72
84
Time Relative to Intramammary Challenge (h)
