# Supplementary material for: The Impact of Intramammary Escherichia coli Challenge on Liver and Mammary Transcriptome and Cross-Talk in Dairy Cows during Early Lactation Using RNAseq
Source: PLoS One. 2016 Jun 23;11(6):e0157480. doi: 10.1371/journal.pone.0157480 (PMC4919052; doi:10.1371/journal.pone.0157480)
Supplement: Supplementary file 3 [file pone.0157480.s002.docx]

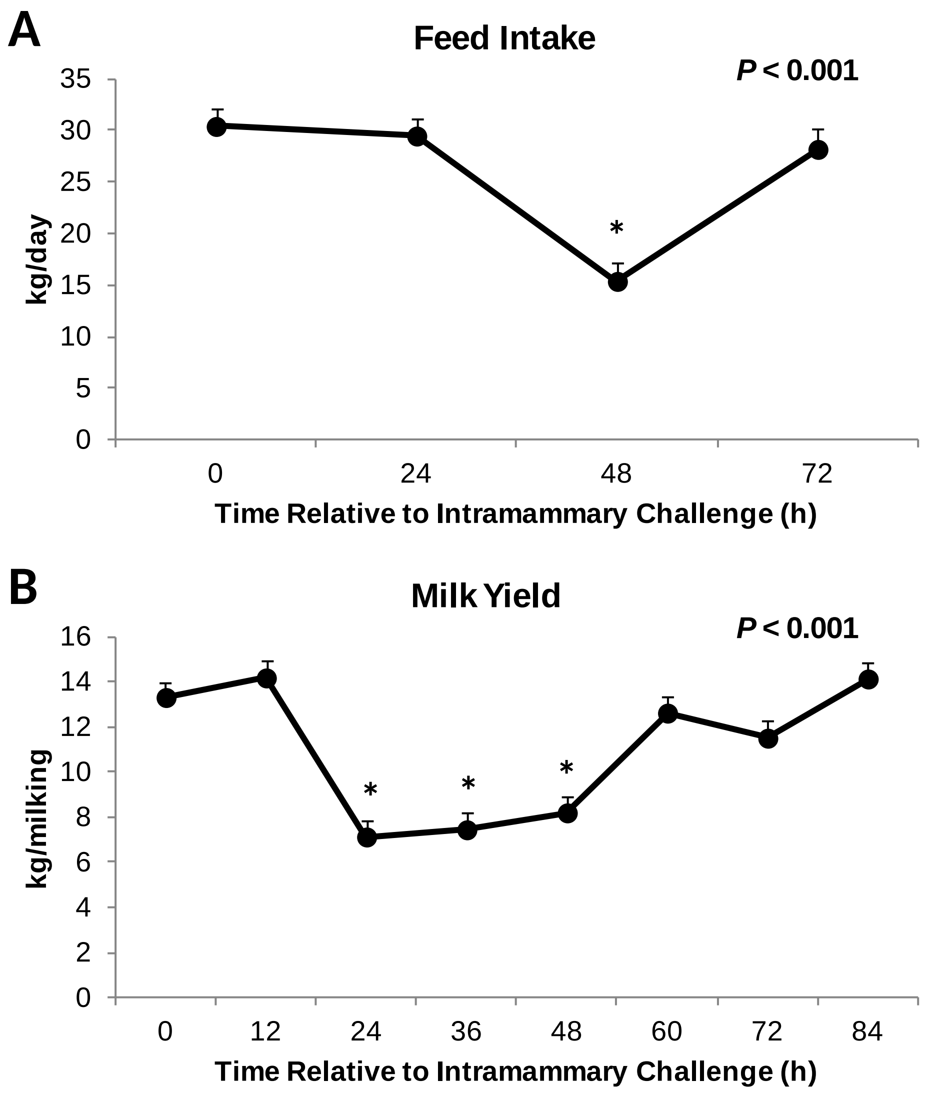
A
Feed Intake
P < 0.001
kg/day
35
30
25
20
15
10
5
0
*
0
24
48
72
Time Relative to Intramammary Challenge (h)
B
Milk Yield
P < 0.001
kg/milking
16
14
12
10
8
6
4
2
0
*
*
*
0
12
24
36
48
60
72
84
Time Relative to Intramammary Challenge (h)
