# Supplementary material for: The Impact of Intramammary Escherichia coli Challenge on Liver and Mammary Transcriptome and Cross-Talk in Dairy Cows during Early Lactation Using RNAseq
Source: PLoS One. 2016 Jun 23;11(6):e0157480. doi: 10.1371/journal.pone.0157480 (PMC4919052; doi:10.1371/journal.pone.0157480)
Supplement: Supplementary file 4 [file pone.0157480.s003.docx]

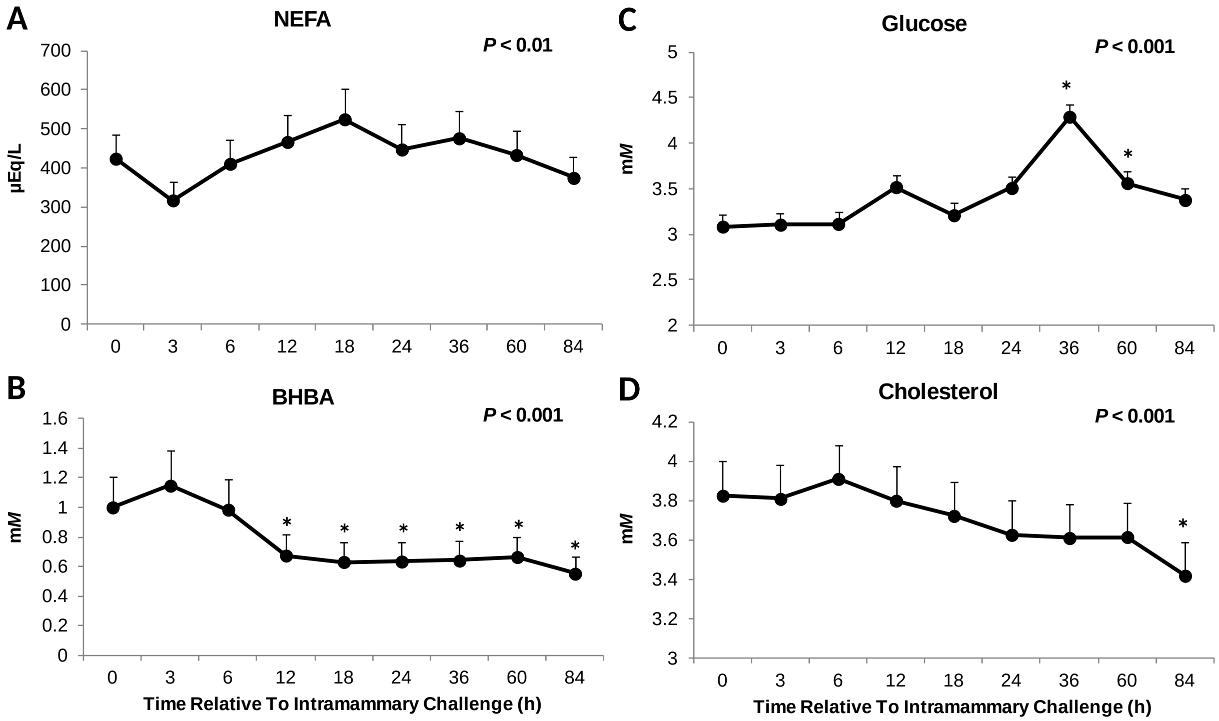

A
NEFA
P < 0.01
µEq/L
700
600
500
400
300
200
100
0
0
3
6
12
18
24
36
60
84
C
Glucose
P < 0.001
mM
5
4.5
4
3.5
3
2.5
2
*
*
0
3
6
12
18
24
36
60
84
B
BHBA
P < 0.001
mM
1.6
1.4
1.2
1
0.8
0.6
0.4
0.2
0
*
*
*
*
*
*
0
3
6
12
18
24
36
60
84
Time Relative To Intramammary Challenge (h)
D
Cholesterol
P < 0.001
mM
4.2
4
3.8
3.6
3.4
3.2
3
*
0
3
6
12
18
24
36
60
84
Time Relative To Intramammary Challenge (h)
